# Supplementary figures and images for: Challenges for funders in monitoring compliance with policies on clinical trials registration and reporting: analysis of funding and registry data in the UK
Source: BMJ Open. 2020 Feb 17;10(2):e035283. doi: 10.1136/bmjopen-2019-035283 (PMC7045207; doi:10.1136/bmjopen-2019-035283)

SUPPLEMENTARY FILE 1: POLICY TIMELINE

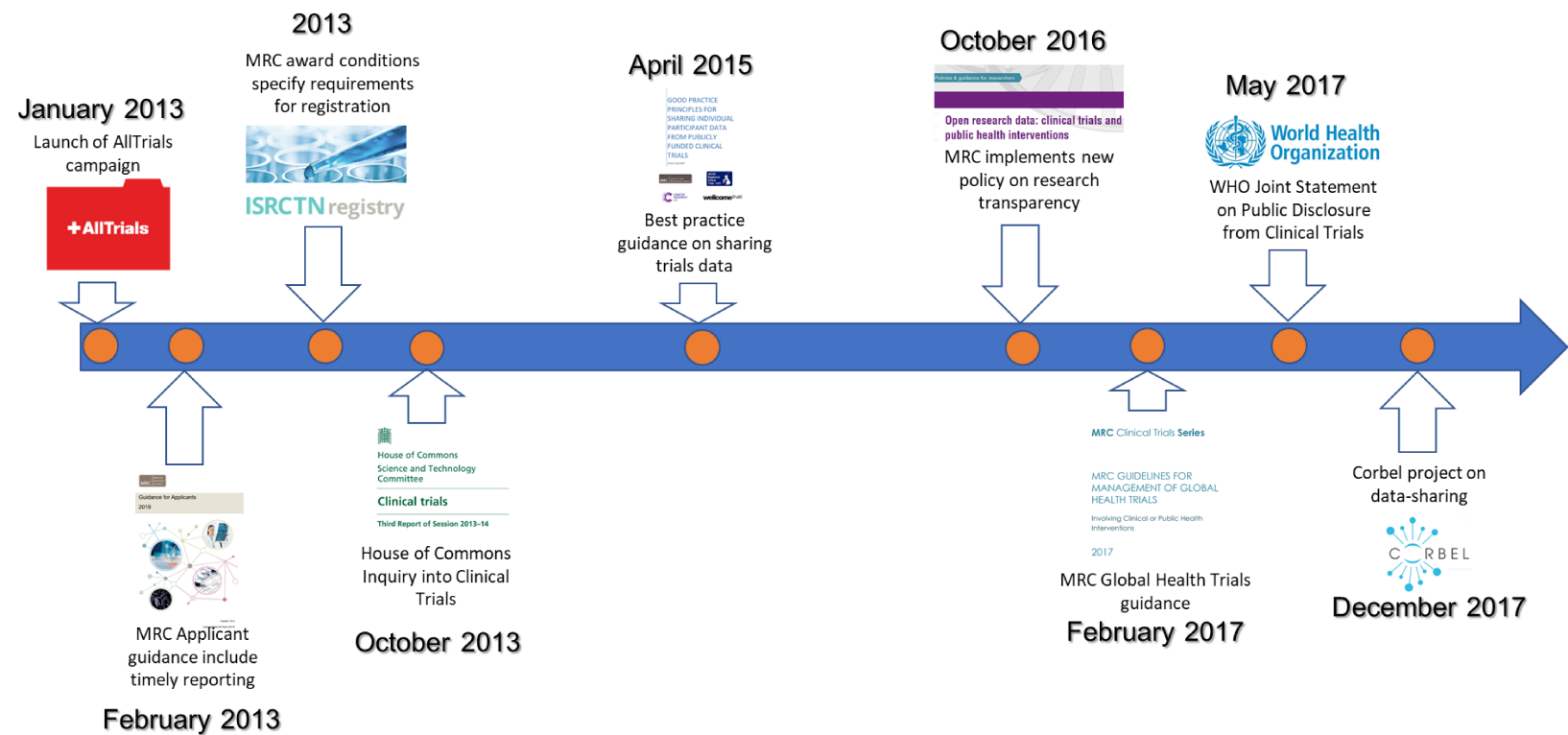

Supplement: Supplementary data [file bmjopen-2019-035283supp001.pdf]
