# Supplementary material for: Challenges for funders in monitoring compliance with policies on clinical trials registration and reporting: analysis of funding and registry data in the UK
Source: BMJ Open. 2020 Feb 17;10(2):e035283. doi: 10.1136/bmjopen-2019-035283 (PMC7045207; doi:10.1136/bmjopen-2019-035283)
Supplement: Supplementary data [file bmjopen-2019-035283supp002.pdf]

SUPPLEMENTARY FILE 2: SEARCH STRATEGY

Original Search Strategy:

1. Grant application/proposal classification includes any of the following keywords:
   a. 'MRC Trial'
   b. 'Clinical Trial'
   c. HRCS Research Activities AND Classification Name begins with [relevant classification code inserted]
2. Keyword search of 'Clinical Trial' on:
   a. Organisation or Department Name
   b. Joint Funder name
   c. Funding Scheme or Call
   d. Grant application:
      i. Technical Summary,
      ii. Lay Summary,
      iii. Impact Summary,
      iv. Aims and Objectives,
      v. Title,
      vi. Keywords.
3. Grant application submitted to:
   a. Board Science Late Phase Trials
   b. Board Science Methodology Research Panel

Concepts and Keywords used in the Revised Search Strategy (on all database fields):

1. Concept: Clinical Trials
   - Clinical Trials
   - Controlled Clinical Trials
   - Randomized Controlled Clinical Trials
   - Randomized Controlled Trials
   - Randomized Clinical Trials
2. Concept: Protocols
   - Clinical Protocols
   - Clinical Research Protocols
   - Treatment Protocols
3. Concept: Trials Monitoring
   - Clinical Trials Data Monitoring Committees
   - Data Monitoring Committees
   - Data and Safety Monitoring Boards
   - Safety Monitoring Boards
   - Phase 1 Clinical Trials
4. Concept: Phase I Trials
   - Phase I Clinical Trials
   - Phase 1 Clinical Trials
   - FDA Phase I Evaluation Studies
   - FDA Phase 1 Evaluation Studies
   - Human Microdosing Trials
   - FDA Phase 1 Drug Evaluation
   - FDA Phase I Drug Evaluation
5. Concept: Phase II Trials

- Phase II Clinical Trials
- FDA Phase II Evaluation Studies
- FDA Phase 2 Evaluation Studies
- FDA Phase II Drug Evaluation
- FDA Phase 2 Drug Evaluation

6. Concept: Phase 3 Clinical Trials
    - Phase 3 Clinical Trials
    - Phase III Clinical Trials
    - FDA Phase III Evaluation Studies
    - FDA Phase 3 Evaluation Studies
    - FDA Phase III Drug Evaluation
    - FDA Phase 3 Drug Evaluation
7. Concept: Phase 4 Clinical Trials
    - Phase 4 Clinical Trials
    - Phase IV Clinical Trials
    - FDA Phase IV Drug Evaluation
    - FDA Phase 4 Evaluation Studies
    - FDA Phase IV Evaluation Studies
    - FDA Phase 4 Drug Evaluation
8. Concept: Cohort Studies
    - Cohort studies
    - Closed Cohort Studies
    - Incidence Studies
    - Cohort Analysis
    - Historical Cohort Studies
    - Concurrent Studies
9. Concept: Control Groups
    - Control Groups
10. Concept: Crossover Design
    - Crossover Design
    - Cross-Over Trials
    - Crossover Trials
    - Cross-Over Design
    - Crossover Studies
11. Concept: Double-Blind Method
    - Double-Blind Method
    - Double-Masked Method
    - Double-Blind Study
    - Double-Masked Study
12. Concept: Feasibility Studies
    - Feasibility Studies
13. Concept: Informed Consent
    - Informed Consent
14. Concept: Intervention Studies
    - Intervention Studies
15. Concept: Mass Screening
    - Mass Screening
16. Concept: Multicenter Studies
    - Multicenter Studies
    - Multicenter Trials

17. Concept: Patient Compliance
    - Patient Compliance
    - Patient Non-Adherence
    - Patient Non-Compliance
    - Patient Nonadherence
    - Patient Cooperation
    - Patient Adherence
    - Patient Noncompliance
18. Concept: Placebos
    - Placebos
    - Sham Treatment
19. Concept: Research Design
    - Data Reporting
    - Research Technics
    - Data Adjustment
    - Research Proposal
    - Data Quality
    - Matched Groups
    - Research Techniques
    - Experimental Designs
    - Problem Formulation
    - Research Methodology
    - Scoring Methods
    - Error Sources
20. Concept: Single-Blind Method
    - Single-Blind Study
    - Single-Masked Method
    - Single-Masked Study
21. Concept: Investigational Therapies
    - Innovative Therapies
    - Experimental Therapies
    - Investigational Therapies
    - Investigational Treatments
