# Supplementary material for: Challenges for funders in monitoring compliance with policies on clinical trials registration and reporting: analysis of funding and registry data in the UK
Source: BMJ Open. 2020 Feb 17;10(2):e035283. doi: 10.1136/bmjopen-2019-035283 (PMC7045207; doi:10.1136/bmjopen-2019-035283)
Supplement: Author's manuscript [file bmjopen-2019-035283.draft_revisions.pdf]

BMJ Open is committed to open peer review. As part of this commitment we make the peer review history of every article we publish publicly available.

When an article is published we post the peer reviewers' comments and the authors' responses online. We also post the versions of the paper that were used during peer review. These are the versions that the peer review comments apply to.

The versions of the paper that follow are the versions that were submitted during the peer review process. They are not the versions of record or the final published versions. They should not be cited or distributed as the published version of this manuscript.

BMJ Open is an open access journal and the full, final, typeset and author-corrected version of record of the manuscript is available on our site with no access controls, subscription charges or pay-per-view fees (http://bmjopen.bmj.com).

If you have any questions on BMJ Open's open peer review process please email info.bmjopen@bmj.com

BMJ Open

Challenges for funders in monitoring compliance with policies on clinical trials registration and reporting: analysis of funding and registry data.

Journal:	*BMJ Open*
Manuscript ID	bmjopen-2019-035283
Article Type:	Original research
Date Submitted by the Author:	28-Oct-2019

Complete List of Authors:	Knowles, Rachel; Medical Research Council,<br>Ha, Kam Pou; Medical Research Council<br>Mueller, Julia ; Manchester University, Population Health, Health Services Research and Primary Care<br>Rawle, Frances; Medical Research Council<br>Parker, Rosa; Medical Research Council

Keywords:	Clinical trials < THERAPEUTICS, STATISTICS & RESEARCH METHODS, AUDIT

SCHOLARONE™
Manuscripts

I, the Submitting Author has the right to grant and does grant on behalf of all authors of the Work (as defined in the below author licence), an exclusive licence and/or a non-exclusive licence for contributions from authors who are: i) UK Crown employees; ii) where BMJ has agreed a CC-BY licence shall apply, and/or iii) in accordance with the terms applicable for US Federal Government officers or employees acting as part of their official duties; on a worldwide, perpetual, irrevocable, royalty-free basis to BMJ Publishing Group Ltd ("BMJ") its licensees and where the relevant Journal is co-owned by BMJ to the co-owners of the Journal, to publish the Work in this journal and any other BMJ products and to exploit all rights, as set out in our licence.

The Submitting Author accepts and understands that any supply made under these terms is made by BMJ to the Submitting Author unless you are acting as an employee on behalf of your employer or a postgraduate student of an affiliated institution which is paying any applicable article publishing charge ("APC") for Open Access articles. Where the Submitting Author wishes to make the Work available on an Open Access basis (and intends to pay the relevant APC), the terms of reuse of such Open Access shall be governed by a Creative Commons licence – details of these licences and which Creative Commons licence will apply to this Work are set out in our licence referred to above.

Other than as permitted in any relevant BMJ Author's Self Archiving Policies, I confirm this Work has not been accepted for publication elsewhere, is not being considered for publication elsewhere and does not duplicate material already published. I confirm all authors consent to publication of this Work and authorise the granting of this licence.

For peer review only

TITLE PAGE

Title: Challenges for funders in monitoring compliance with policies on clinical trials registration and reporting: analysis of funding and registry data.

Short title: Challenges for funders in monitoring compliance with policies on clinical trials registration and reporting.

Authors: Rachel L Knowles, Kam Pou Ha, Julia Mueller, Frances Rawle, Rosa Parker

Corresponding author:

Dr Rachel L Knowles

Medical Research Council (UK Research and Innovation)

58 Victoria Embankment

London EC4Y 0DS

United Kingdom

Email: rachel.knowles@mrc.ukri.org

Telephone: +44 (0)20 7395 2256

ORCiD: 0000-0002-5490-7682

Author affiliations:

Rachel L Knowles, Medical Research Council (UK Research and Innovation), London, United Kingdom

Kam Pou Ha, Medical Research Council (UK Research and Innovation), London, United Kingdom

Julia Mueller, Medical Research Council (UK Research and Innovation), London, United Kingdom (ORCiD: 0000-0002-4939-7112)

Frances Rawle, Medical Research Council (UK Research and Innovation), London, United Kingdom

Rosa Parker, Medical Research Council (UK Research and Innovation), London, United Kingdom

Word count: 4425 words (main text); abstract 274 words; 52 references; 4 figures; 2 tables

Keywords

clinical trials; registration of trials; disclosure of results; publication bias; outcome reporting; transparency; open science

For peer review only - http://bmjopen.bmj.com/site/about/guidelines.xhtml

ABSTRACT

Objectives:

To evaluate compliance by researchers with funder requirements on clinical trials transparency, including identifying key areas for improvement; to assess the completeness, accuracy and suitability for annual compliance monitoring of the data routinely collected by a research funding body.

Design:

Descriptive analysis of clinical trials funded between February 2011 and January 2017 against funder policy requirements.

Setting:

Public medical research funding body in the United Kingdom.

Data sources:

Relevant clinical trials were identified from grant application details, post-award grant monitoring systems and the ISRCTN registry.

Main outcome measure:

Proportion of all MRC-funded clinical trials that were (a) registered in a clinical trials registry and (b) publicly reported summary results within 2 years of completion.

Results:

There were 175 grants awarded that included a clinical trial and all trials were registered in a public trials registry. Of 62 trials completed for over 24 months, 42 (68%) had publicly reported the main findings by 24 months after trial completion; 18 of these achieved this within 12 months of completion. Eleven (18%) trials took longer than 24 months to report and 9 (15%) completed trials had not yet reported findings. Five datasets were shared with other researchers.

Conclusions:

Compliance with the funder policy requirements on trials registration was excellent. Reporting of the main findings was achieved for most trials within 24 months of completion, however the number of unreported trials remains a concern and should be a focus for future funder policy initiatives. Identifying trials from grant management and grant monitoring systems was challenging therefore funders should ensure investigators reliably provide trial registries with information and regularly update entries with details of trial publications and protocols.

For peer review only - http://bmjopen.bmj.com/site/about/guidelines.xhtml

ARTICLE SUMMARY

Strengths and limitations of this study

- This study investigated the feasibility, and highlighted the challenges, for a public funder of implementing an annual audit of compliance by researchers with a clinical trials transparency policy.
- Using grant applications, annual grant monitoring and trials registry data, the proportion of all funded clinical trials that were (i) publicly registered and (ii) for which results were publicly reported was calculated for a six-year period.
- Study strengths included the completeness of the dataset and careful validation of registration and publication details.
- Analyses were limited by the small number of trials per year.
- Improvements in systems for compliance monitoring could make better use of data within trials registries and include reminders to researchers as part of the monitoring process.

For peer review only

For peer review only - http://bmjopen.bmj.com/site/about/guidelines.xhtml

INTRODUCTION

It is estimated that around 1 million clinical trials have been carried out since 1948, when the UK Medical Research Council (MRC) conducted a landmark randomised controlled trial on streptomycin in pulmonary tuberculosis.[1] Although responsibility for funding Phase 3 clinical trials in the UK transferred to the National Institute for Health Research (NIHR) in 2006, the MRC, as part of UK Research and Innovation, continues to directly fund early phase clinical trials, Phase 3 global health trials, other clinical and public health intervention studies, and to provide underpinning funding for trials methodology research and clinical trials units.

Serious concerns have been raised about the failure by researchers to report findings from a high proportion of clinical trials, as well as the selective reporting of 'positive' findings.[2-6] This represents not only a loss of valuable research findings but may lead to potential overestimation of the benefits of new treatments, with the risk that ineffective or harmful drugs are released to market. [3 7] Public trials registers, which facilitate the prospective registration of clinical trials, play a key role in reducing duplication of reporting and publication bias[8] and the International Consortium of Medical Journal Editors (ICMJE) requires prospective registration of trials as a condition of publication in its member journals.[9] The World Health Organization (WHO) has established an International Clinical Trials Registry Platform (ICTRP), which collects information on ongoing and completed trials from a network of primary registries across the world and provides a single point of public access.[10 11]

The AllTrials[12] campaign was established to actively promote clinical trials transparency *"All trials registered All trials reported"*, and funder signatories to the AllTrials petition, such as the UK MRC, subsequently strengthened requirements for grant recipients to prospectively register clinical trials and publicly disseminate results (Supplementary File 1). Despite this, a UK House of Commons Science and Technology Committee (STC) Inquiry into Research Integrity: Clinical Trials Transparency in 2018 concluded that *"registration is not yet universal … and reported outcomes do not always align with the original study proposal".* The Committee strongly recommended that compliance with reporting requirements should be monitored by funders and regulators and published at individual trial level.[13] This call for improved monitoring of compliance with registration and reporting policies echoed the WHO *Joint statement on public disclosure of results from clinical trials* to which 21 funding agencies have now signed up.[14]

As a founder signatory to the WHO joint statement, the MRC committed to developing ongoing monitoring of compliance with its *Policy on Open Research Data from Clinical Trials and Public Health Intervention Studie*s published in October 2016[15] (Table 1). An initial review of research grants awarded and active over the five-year period 01 February 2011 to 31 January 2016 identified 107 clinical trials of which 101 (94%) were registered in a public trials registry. From 40 completed trials, 33 (82%) reported at least one publication.[16]

In this paper, we present a subsequent evaluation undertaken in 2018 that included MRC funding for awards involving a clinical trial up to 31 January 2017. Our aims were (1) to evaluate compliance by Principal Investigators (PIs; lead researcher or Grant Holder) with MRC's clinical transparency requirements, including after introduction of the new policy, and to identify key areas for improvement, and (2) to assess data completeness and accuracy as a basis for the development of annual compliance monitoring.

For peer review only - http://bmjopen.bmj.com/site/about/guidelines.xhtml

Table 1: Summary of MRC policy requirements (October 2016)*

**Registration**
1. Prospective registration of every MRC-funded clinical trial in the ISRCTN trials registry* prior to recruitment of first participant;<br>*(*other WHO primary registries accepted if prior agreement with the MRC)*
2. Provision of the registry number to the MRC within 12 months of registration;
3. Reviewing (and updating if appropriate) the registry entry at least once per year until the trial has reported main results;
**Reporting**
4. Public report of the trial protocol – there should be a link to this from the registry entry (the protocol may be added as a supplementary document in the registry entry);
5. Public report of the trial's main results in a timely manner (usually within 12 months); there should be a link to the open accessible report/publication from the registry entry (and/or a report may be added as a supplementary document in the registry entry);
**Data Sharing**
1. Preparation of the trial dataset for sharing with external researchers is encouraged; researchers are expected to provide details of any datasets/databases created as part of an award in their annual report to the MRC.

* This table summarises requirements from the MRC policy[15]

METHODS

Data sources

The initial step was to compile a single monitoring dataset that included all awards made by the MRC during the audit period from 01 February 2011 until 31 January 2017, which included a clinical trial meeting the WHO definition: *"any research study that prospectively assigns human participants or groups of humans to one or more health-related interventions to evaluate the effects on health outcomes."*[17] The dataset included research grants, fellowship awards, global health trials, and any developmental clinical study award in which preclinical work was followed by a clinical trial. Trials funded through MRC Research Unit and MRC/University Unit programmes to Clinical Trials Units or joint-funding not directly managed by the MRC, for example the NIHR Efficiency and Mechanism Evaluation (EME) programme, were excluded. Information was extracted from four sources (Table 2): the MRC grants application and award database; the annual output, outcome and reporting system (Researchfish®); a listing of joint-funded global health trials for which the MRC had oversight; and an external dataset provided by the ISRCTN registry.[18]

Basic keyword searches (Supplementary File 2) of MRC grants application and annual monitoring databases for the 2017 audit yielded 373 funded awards that potentially included a trial (Figure 1), for 112 of which a trial registry number had been reported to the MRC. Where trial registry numbers had not been reported, searches were undertaken to complete missing entries. The grant reference, study title, acronym and PI name were entered into the three clinical trials registries most commonly used for UK research, the ISRCTN registry[18], ClinicalTrials.gov[19] and EU Clinical Trials Registry (EUCTR)[20], followed by an online web search using these terms. Of 288 studies with a registry entry,

For peer review only - http://bmjopen.bmj.com/site/about/guidelines.xhtml

124 were excluded as they did not meet the WHO clinical trial definition. Of the remaining 85 studies, 60 were excluded after review as not including a clinical trial while 25 were identified as potentially including a clinical trial. These PIs were contacted for further information; 15 had not progressed beyond preclinical research, the registry number was provided for nine trials, and one trial was unregistered however the research team registered it retrospectively after being contacted.

Following the collection of annual reports (using Researchfish®) from researchers in February 2018, the MRC dataset was updated (Figure 1) and 16 studies were excluded due to new information. Eleven new awards made between 01 February 2016 and 31 January 2017 were added, as well as five studies that had progressed from preclinical research to a clinical trial during this period. Global health trials with partnership funding undergo additional governance monitoring by the MRC and this list yielded 28 additional trials not previously included in the analysis.

A search of the independent ISRCTN registry dataset yielded 132 entries in which a trial had started on or after 01 February 2011 and the MRC was named as the funder or sponsor; 90 trials were not eligible for inclusion (due to information on funder/sponsor being incorrect), 12 trials matched those already identified, and an additional 30 previously unidentified trials were added to the review dataset.

In total, 175 awards made by the MRC between 01 February 2011 and 31 January 2017 were identified as including a clinical trial and were eligible for inclusion in the 2018 review dataset.

Prior to commencing the review, project titles and abstracts of all awards were reviewed to determine whether they included a clinical trial and classified as a *trial*, *not a trial*, *trial not started* (preclinical), or '*unclear*'. If a study was classified 'unclear' the full grant application was reviewed and a consensus classification agreed by two reviewers. PIs were contacted if uncertainty remained about whether the award included a clinical trial, or if the registry numbers or main results publications could not be identified.

Evaluating the monitoring process: data completeness and accuracy

Each source contributing to the review dataset was individually assessed to determine the proportion of the total dataset that was ascertained and the reasons for any trials not being identified.

To assess accuracy of the review dataset, specific data items were compared between the MRC award information and the registry entry for each trial; this included checking MRC grant reference numbers, award/trial dates, PI name and research organisation, and publications.

Evaluating researcher compliance with policy requirements

Analyses of compliance with the MRC *Policy on Open Research Data from Clinical Trials and Public Health Intervention Studies* (2016) included the number and percentage of:

1. *all awards* that were registered in a public trials registry and percentage registered in the ISRCTN (as required by the policy) or another registry;
2. *all registry entries* that had a link to the study protocol or publication/report;
3. *all completed awards* that had publicly reported main, or summary, results;
4. *all awards* that reported creating a dataset for sharing beyond the study team/collaborators.

Changes in registration and reporting over time were investigated to explore the impact of the MRC introducing requirements for trials registration in 2013[21], and implementation of the new policy in 2016.[15]

For peer review only - http://bmjopen.bmj.com/site/about/guidelines.xhtml

Table 2 Sources of data for monitoring

Database	Purpose of database	Data entered by:	Data collected (relevant to monitoring)	Search strategy
**MRC Grants Application and Award database**	MRC application management and monitoring. Data is publicly available in UKRI Gateway to Research.[22]	Applicants	Data entered as part of funding application and award process.<br>1. Award start and end dates<br>2. PI contact details<br>3. Study title and summary<br>4. Amount of award	**Automated** keyword search in title, abstract, study summary;
**MRC annual monitoring of awards (Researchfish®)**	MRC monitoring of activity and outputs from research funding. Data collected via the Researchfish® platform. Data is publicly available in UKRI Gateway to Research.[22]	Named PI receiving funding	Annual report/update to the MRC on 'live' or recently ended awards every February. Includes:<br>1. Clinical trial 'flag', i.e. trial associated with award<br>2. Trials registry number<br>3. Publications and other outputs<br>4. Datasets created/shared	**Automated** keyword search in title, abstract, study summary, outputs, publication fields; **Automated** search for clinical trials 'flag', entry for registry number
**MRC governance monitoring of global health trials**	MRC governance of global health clinical trials.<br>Data not publicly available.	MRC staff	Information entered for governance and oversight<br>1. Record of Trial Steering Committee TSC dates<br>2. Changes to trial start and end dates	**Manual** search of study summary or notes to confirm study design and identify trials;
**ISRCTN registry[18]**	Publicly accessible and searchable database of individual clinical trials. Trial information is regularly submitted to WHO ICTRP portal where it is publicly available.[23]	Research team	Data entered at registration then updated or amended by research team as required.<br>1. Trial start and end dates<br>2. Contact person<br>3. Registration date<br>4. Links to protocol and main results<br>5. Trial funder<br>6. Trial sponsor<br>7. Date entry last updated	**Automated** search for Medical Research Council or MRC in funder and sponsor fields;

For peer review only - http://bmjopen.bmj.com/site/about/guidelines.xhtml

As information on award outputs is submitted annually by PIs using the Researchfish® system, there can be a delay in reporting of registration or publications of up to 12 months. Therefore, analysis of registration was restricted to awards in which the trial had started at least 12 months prior to the analysis data extract being taken (01 February 2018) and analysis of publications was restricted to studies in which the funding award or trial end date (the later of these dates) was at least 12 months before the data extract (referred to as *completed awards*).

Research publications or reports were only considered to report the main results from the trial if they (i) were defined in the ISRTCN registry entry as including the main results, (ii) included information in the publication stating that they reported the main results, or (iii) were confirmed by the PI to report the main results. If a trial end date was after the funding award end date, then trials were considered completed on the trial end date and time to publication was calculated using this date.

RESULTS

Completeness of trials data from different sources

The contribution that each of the four data sources (Table 2) made to the final review dataset was assessed. The original search strategy (Supplementary File 2) applied keywords to specific fields in the MRC grants application and annual monitoring databases but identified only 117 (67%) of 175 awards in the review dataset that included a trial. Revised keyword searches (Supplementary File 2) were then developed and these identified 913 studies, which were categorised by likelihood of including a clinical trial: category 1=highly likely to include a clinical trial [n=332]; category 2=a clinical study or trial [n=274]; 3=patient or cohort study [n=252]; categories 4=clinical trial methods [n=32]; category 5=intervention study [n=23]). The revised searches had higher sensitivity than the original search, identifying 149 (85% of 175) awards as likely to include a clinical trial in the final review dataset, however they had poor positive predictive value as only 20% of retrieved awards were found to include clinical trials.

Assessment of annual reports to the MRC also found that the trials registry number was not consistently reported and only 54 (29% of 175) awards were identified as including a trial via this data.

The 28 trials identified from global trials governance monitoring were not identified by the original keyword searches of MRC databases. They were also not identified through the ISRCTN registry search, mainly because they received partnership funding and the MRC was not the primary funder.

The ISRCTN registry yielded 30 (17% of 175) trials that would otherwise have been missed. However, a further 90 (68% of the 132) studies identified by the ISRCTN search were excluded as further checks of the registry entry showed that the trial either did not receive MRC funding or the study was not a clinical trial. For example, inaccurate entries could occur because one member of the trial team was employed in an MRC unit or received an MRC fellowship for a different project. Therefore, both the search strategy and the accuracy of the registry entries presented significant challenges for accurate identification of relevant trials.

Accuracy of publicly available trials data

Review of study abstracts by reviewers, and where necessary full applications, enabled exclusion of most studies that did not include a clinical trial. However it was necessary to contact PIs about 55 studies, from which 16 studies were subsequently excluded from the review dataset (Figure 1).

For peer review only

Information on MRC awards, including award reference, project titles, summary and technical summary, PI, research organisations and award dates and values are published in UK Research and Innovation (UKRI) Gateway to Research.[22] Trials registry entries usually record trial dates, the PI's name, a contact person (who may also be the PI), and the organisation acting as the trial sponsor. When Gateway to Research and registry entries were compared, the organisation holding the MRC award was named in 159 (91%) registry entries whereas the PI of the MRC grant was only named in 114 (65%) entries. The MRC reference number was provided in 60 (34%) registry entries and a further 67 (38%) entries named the MRC as a funder, while 48 (27%) did not acknowledged MRC funding. The dates of the trial in the registry entry and the MRC award dates matched completely in only five trials, as in most cases awards start some time before participant recruitment. The difference between these dates was greater where awards included preclinical studies prior to commencement of the clinical trial.

Of 3420 journal articles reported in Researchfish® by researchers as outputs from the 175 MRC awards involving a clinical trial between 2011 and 2017, only six of these were listed as the 'main results' publications in the ISRCTN registry.

Trials registration

All 175 (100%) trials were registered in a public trials registry; 96 (55%) of these in the ISRCTN registry and 79 (45%) with other registries, such as ClinicalTrials.gov and EUCTR. One-quarter (n=43) were registered in more than one registry. Registry entries for 84 (76%) of the 111 trials ongoing in March 2019 had been updated since 01 January 2017. The trial protocol, or an electronic link to the protocol, was provided in only 63 (36% of 175) registry entries.

The percentage of MRC trials registered in the ISRCTN registry varied over time but there was a decrease in trials registering retrospectively (Figure 2). However the introduction of requirements in award conditions in 2013 to use the ISRCTN registry, and to acknowledge MRC funding, has yet to have a discernible impact.

Public reporting of main trial results

Sixty-two awards/trials were completed before 01 February 2017, of which 42 (68%) publicly reported main results, or posted results in a registry, within 24 months of award/trial end. Only 18 (29% of 62) trials reported within 12 months of award/trial end. A further 11 (18% of 62) completed trials reported main results more than 24 months after award/trial end. Nine trials had not reported results (at March 2019).

Of 62 completed awards, 40 (65%) had a link to the main results or posted results in the registry (links=40; results posted=4; both were added for some trials).

The time taken to report results, for the 53 completed and reported trials, ranged from 8 months prior to 56 months after award/trial end, and the majority reported between six and 24 months after award/trial end (Figure 3).

The PIs for the nine unreported trials were contacted for further information. Seven reported that papers were progressing and publication was expected within the next 12 months. The period since trial completion ranged from 24 to 43 months; various reasons for delay were highlighted, including absence of a key researcher, negative findings or early trial termination. One award had included findings in a press release and symposium abstract; these were insufficiently detailed to be considered main results.

For peer review only - http://bmjopen.bmj.com/site/about/guidelines.xhtml

There was an increase in the percentage of trials reporting results before 24 months in 2016-2017 compared with most previous years; in contrast, the percentage of trials that reported within 12 months changed little over time (Figure 4). In recent years, the percentage of unreported trials increased, reflecting the higher number of trials ending in these years as well as delays in reporting. Based on previous evidence, it is likely that the total number of unreported trials will reduce further with late publications.

Data-sharing

PIs reported creating 18 datasets for sharing with other researchers as an output from their clinical trial data, representing 10% of all 175 trials. Five of these datasets had been shared with researchers outside the original study team, one had been deposited in a repository for reuse by other researchers, and one was available through a disease registry.

DISCUSSION

As a signatory to AllTrials[12] and the WHO Joint Statement[14], the MRC has made a commitment to the implementation of regular monitoring of researchers' compliance with the registration and reporting requirements for clinical trials set out in its *Policy on Open Research Data from Clinical Trials and Public Health Intervention Studies (2016)*.[15] Although the review found that all 175 trials funded by the MRC between 2011 and 2017 were registered, only half were registered in the WHO primary registry specifically named in the MRC award conditions and researchers often provided different information in the registry entry to that held by the funder. Summary results were reported within two years of completion for almost 70% of trials; most through academic papers but two-thirds also added links to these results in the trials registry entry. Despite this the percentage of trials that had been completed for over two years but had failed to report was unacceptably high (15%). This underlines the need for renewed joint efforts by the MRC to address the barriers to timely publication that were identified, which included delays in publishing negative findings after early trial termination, and research staff absence or turnover.

Registration

MRC-funded researchers are required to register trials in the ISRCTN registry because it permits registration of the wide range of study designs supported by the MRC and, as a WHO primary registry, contributes regularly to the International Clinical Trials Registry Platform (ICTRP)[24] thus maximising access to trials information. MRC policy explicitly states that researchers must add trial protocols and publications, as well as regularly reviewing and updating registry entries to ensure these remain accurate. An important positive finding was the decrease in the proportion of trials registering retrospectively, a trend that has also been noted by other authors and attributed to rising awareness among researchers of the need to register their trials.[25-27] However, our review is consistent with previous reports[28 29] that have highlighted inaccurate and incomplete registry entries as a significant cause for concern. This suggests that registries should review their guidance and controls around data entry by researchers to improve the consistency and accuracy of trial records. Disappointingly, a high proportion of trials in our review were not registered in the ISRCTN registry, and it was noticeable that information in many registry entries was discrepant with that held by the MRC. Furthermore only one-third of registry entries included a link to the trial protocol, despite early protocol publication being key to minimising publication bias and selective reporting of trial findings.[14 30] These findings suggest that researchers do not fully appreciate their responsibilities with regard to clinical trials transparency, specifically the need to regularly update registry entries so that they provide accurate and complete information to participants, the public and the research community. We also observed that the responsibility for complying with clinical trials transparency

For peer review only - http://bmjopen.bmj.com/site/about/guidelines.xhtml

requirements falls largely upon the shoulders of individual researchers and would therefore strongly endorse the call by Goldacre et al[31] for trial sponsors and research organisations which host trials to take greater responsibility for promoting and monitoring transparency, providing more administrative support, and raising awareness and compliance among researchers.

Reporting results

Funder signatories to the WHO Joint Statement[14] committed to developing policies on timely publication, including an indicative timeframe of 24 months for publication in a journal and working towards a timeframe of 12 months for reporting summary results in a registry. Current MRC policy includes an expectation that main trial results will be reported "usually" within 12 months, but this is not mandated. EU and US clinical trials legislation mandate the posting of trial results by 12 months after trial end[32 33] although this is often not achieved[31 34], while other non-commercial funders have mandated timeframes ranging from 12 to 24 months after trial completion.[7 35] Our review confirms 24 months as an achievable timeframe for publication in peer reviewed journals for MRC PIs; therefore including a policy requirement for all trials to *either* publish *or* report findings in the registry by 24 months would encourage significant improvement on current practice. We also demonstrated that contacting researchers about reporting was an effective prompt that resulted in submission for publication. This is consistent with the findings of the national audit undertaken by the UK Health Research Authority (HRA)[27] and could be easily integrated into the routine annual review process by funders, registries and regulators. Although trials with null or negative findings often experience longer delays to publication[36 37], there is increasing recognition by journals of the importance of null studies. This is underlined by the development of journals focusing solely on such studies, initiatives such as the recent special supplement of Neurology on the null hypothesis[38], as well as the introduction of the "registered reports" model to some journals.

Data sharing

Funders are increasingly encouraging researchers to make their data available after study completion to maximise the opportunity for health improvement from public investment in research[39], however we found little evidence of trial datasets being prepared or made available for reuse by researchers. In recent years, significant efforts have been made to develop guidance[40-43] and services to enable researchers to share data, including data repositories and online catalogues, independent access review committees, secure platforms for data access and analysis, and data anonymisation tools to reduce disclosure risk.[44-47] There have also been various initiatives to increase academic interest and innovation in data sharing, such as the SPRINT challenge.[48] However, as yet, these incentives to share data do not appear to be sufficiently persuasive and a central concern remains the failure to adequately recognise or 'credit' those who create important data resources for use by other researchers.[49 50]

Sustainable and effective compliance monitoring

Conducting this review demonstrated that deriving a complete trials dataset for annual monitoring currently requires the cross-validation of data extracts from multiple existing sources. No single source contributed more than 85% of the final dataset, nor does any have sufficiently high levels of accuracy to be relied upon as the sole data source for compliance monitoring at present. Inaccuracy and incomplete data was often due to a failure by researchers to maintain and update registry entries as has previously been reported.[29 51] Nevertheless the ISRCTN registry was a reliable source of summary results due to active follow-up of researchers to request information about the main results publications for completed studies. In contrast, although both EUCTR and ClinicalTrials.gov

For peer review only - http://bmjopen.bmj.com/site/about/guidelines.xhtml

provide templates for posting results directly into the registry, only four of the studies in our dataset had done this.

Although keyword searches successfully identified many trials, our review required significant commitment of staff time to perform extensive internet searches, check the accuracy of data, and cross-validate information from different sources. This included visual checks of registry entries and grant applications to ensure that studies were eligible trials and that information was complete; this was only possible with the help of Policy Interns undertaking placements as part of an MRC-funded PhD studentship. Importantly over 30% of PIs had to be contacted directly one or more times to confirm that a study had progressed beyond the preclinical phase, or to provide registry numbers or publication details. Although initiatives using automated monitoring to identify trials which have failed to report findings have been pursued elsewhere[31 51], it is not currently possible to 'automate' the compliance monitoring process for MRC awards. The challenge of introducing automated methods to monitor trial publications was also highlighted in the HRA national audit of ethical approvals.[27 52] Although it is not feasible for the MRC to commit such a high level of staff and resources to carrying out this manual verification process every year, the dataset created during the current review now reflects the complete clinical trials portfolio and can be supplemented each year with information about new funding awards. The key to further reducing the burden of compliance monitoring for funders and regulators is likely to be greater reliance on registry data, therefore it is essential to focus on strengthening requirements for researchers to maintain accurate, complete and regularly updated registry entries for all ongoing trials.

Strengths and limitations

As with the national audit undertaken by the UK Health Research Authority (HRA)[27], our review was based on a 'complete' denominator, comprising all MRC awards including a clinical trial during the review period, so accurately reflects registration and publication rates. PIs were contacted if there was any uncertainty about whether a study met the WHO definition of a clinical trial and studies that were not required to register under our policy were excluded, for example, non-interventional, genetic or biomarker studies, or studies that failed to progress beyond the preclinical stages.

A limitation of our review dataset was the relatively small number of awards, in particular completed trials, during the period, which had an impact on analyses of publication rates and investigation of trends. We did not attempt to extend the dataset before 2011 as the MRC did not introduce applicant guidance on trials registration until 2013 so this would not have reflected contemporary practice however, in future reviews, additional years of data will be added to the current dataset.

CONCLUSIONS

As the current compliance review mainly included MRC awards before the publication of the new transparency policy, it is perhaps best considered as a reflection of existing practice. However, it will provide a clear benchmark for assessing improvement in clinical trials registration and reporting in future years. This review highlighted some key issues for funders consideration when establishing compliance monitoring including (1) developing the use of registry data as a primary source for compliance monitoring, (2) strengthening funder requirements for registry entries to be maintained, (3) improving identification of funded awards that include trials to ensure a complete denominator for evaluating compliance with registration, and (4) including the research organisation, registry number and award reference number in compliance monitoring reports to allow further details of individual trials to be found in public registries.

For peer review only - http://bmjopen.bmj.com/site/about/guidelines.xhtml

As a funder of a complex clinical trials portfolio, spanning early phase translational clinical trials as well as late phase trials involving behavioural, psychological and public health interventions, the MRC has a keen interest in promoting research transparency across all research involving human participants regardless of study design. An initial step has been to extend the MRC *Policy on Open Research Data from Clinical Trials and Public Health Intervention Studies* (2016) we are now working towards the inclusion of explicit statements about transparency within the data management plans of all funded clinical and public health studies.

For peer review only

Acknowledgements: We would like to thank staff at the ISRCTN registry and within the MRC Evaluation Team for providing the data for the review.

Author contributions: RK**,** RP and FR conceived and designed the study. JM, KPH and RK extracted and analysed data and all authors interpreted the data. RK and RP drafted the manuscript; all authors reviewed and approved the final manuscript. RK is first author and guarantor. This paper is compliant with the STROBE checklist. The corresponding author attests that all listed authors meet authorship criteria and that no others meeting the criteria have been omitted.

Competing interests: All authors have completed the ICMJE uniform competing interest form (available on request from the corresponding author).

Funding: JM and KPH were funded by MRC Policy Internships. This research received no specific grant from any funding agency in the public, commercial or not-for-profit sectors.

Ethical approval: Not required.

Data sharing: The dataset will be made available on the MRC website as a downloadable file.

Transparency: The lead author (RK) affirms that the manuscript is an honest, accurate, and transparent account of the study being reported; that no important aspects of the study have been omitted; and that any discrepancies from the study as planned have been explained.

This is an Open Access article distributed in accordance with the Creative Commons Attribution Non Commercial (CC BY-NC 4.0) license, which permits others to distribute, remix, adapt, build upon this work non-commercially, and license their derivative works on different terms, provided the original work is properly cited and the use is non-commercial. See: http://creativecommons.org/licenses/by-nc/4.0/

Patient and Public Involvement: This research was done without patient or public involvement. Neither patients nor the public were invited to comment on the study design or results, or contribute to the writing of the paper. A lay language summary of the review will be placed on the MRC website to disseminate findings.

For peer review only

REFERENCES

1. Streptomycin treatment of pulmonary tuberculosis. *Br Med J* 1948;2(4582):769-82
2. Song F, Parekh S, Hooper L, et al. Dissemination and publication of research findings: an updated review of related biases. *Health Technol Assess* 2010;14(8):iii, ix-xi, 1-193. doi: 10.3310/hta14080
3. Chalmers I, Glasziou P. Avoidable waste in the production and reporting of research evidence. *Lancet* 2009;374(9683):86-9. doi: 10.1016/S0140-6736(09)60329-9
4. Antes G, Chalmers I. Under-reporting of clinical trials is unethical. *Lancet* 2003;361(9362):978-9. doi: 10.1016/S0140-6736(03)12838-3
5. Dickersin K, Chan S, Chalmers TC, et al. Publication bias and clinical trials. *Control Clin Trials* 1987;8(4):343-53
6. Hahn S, Williamson PR, Hutton JL. Investigation of within-study selective reporting in clinical research: follow-up of applications submitted to a local research ethics committee. *J Eval Clin Pract* 2002;8(3):353-9
7. DeVito NJ, French L, Goldacre B. Noncommercial Funders' Policies on Trial Registration, Access to Summary Results, and Individual Patient Data Availability. *JAMA* 2018;319(16):1721-23. doi: 10.1001/jama.2018.2841
8. Dickersin K, Rennie D. Registering clinical trials. *JAMA* 2003;290(4):516-23. doi: 10.1001/jama.290.4.516
9. De Angelis C, Drazen JM, Frizelle FA, et al. Clinical trial registration: a statement from the International Committee of Medical Journal Editors. *Lancet* 2004;364(9438):911-2. doi: 10.1016/S0140-6736(04)17034-7
10. Gulmezoglu AM, Pang T, Horton R, et al. WHO facilitates international collaboration in setting standards for clinical trial registration. *Lancet* 2005;365(9474):1829-31. doi: 10.1016/S0140-6736(05)66589-0
11. World Health Organization. International Clinical Trials Registry Platform (ICTRP) Primary Registries 2018 [Available from: http://www.who.int/ictrp/network/primary/en/; accessed 08/06/2019.
12. AllTrials. All Trials Registered - All Trials Reported. [Available from: http://www.alltrials.net/find-out-more/; accessed 11/10/2019].
13. House of Commons Science and Technology Committee (2018). 10th report - Research integrity: clinical trials transparency. HC1480.
14. World Health Organization (2017) Joint statement on public disclosure of results from clinical trials. Available from: [https://www.who.int/ictrp/results/ICTRP_JointStatement_2017.pdf?ua=1; accessed 11/10/2019.
15. UK Medical Research Council. (2016) Policy on Open Research Data from Clinical Trials and Public Health Intervention Studies. [Available from: https://mrc.ukri.org/research/policies-and-guidance-for-researchers/open-research-data-clinical-trials-and-public-health-interventions/; accessed 11/10/2019].
16. House of Commons Science and Technology Committee (2018). 10th Report - Research integrity: clinical trials transparency. Written evidence submitted by the Medical Research Council (RES0041).
17. World Health Organization. Health topics: Clinical trials. 2019. [Available from: https://www.who.int/topics/clinical_trials/en/; accessed 11/10/2019].
18. ISRCTN Registry. [Available from: https://www.isrctn.com/; accessed 11/10/2019].
19. ClinicalTrials.gov. 2019 [Available from: https://clinicaltrials.gov/; accessed 11/10/2019].
20. About the EU Clinical Trials Register. 2019 [Available from: https://www.clinicaltrialsregister.eu/about.html; accessed 11/10/2019].

For peer review only - http://bmjopen.bmj.com/site/about/guidelines.xhtml

21. House of Commons Science and Technology Committee. (2013) Seventh Special Report. Clinical trials: Medical Research Council Response to the Committee's Third Report of Session 2013-14. Appendix 1.
22. UK Research and Innovation. Gateway to Research. [Available from: https://www.gtr.ukri.org/; accessed 08/06/2019].
23. World Health Organization. International Clinical Trials Registry Platform (ICTRP). [Available from: www.who.int/ictrp; accessed 08/06/2019].
24. World Health Organization. International Clinical Trials registry Platform (ICTRP) Primary Registries. [Available from: https://www.who.int/ictrp/network/primary/en/; accessed 11/10/2019].
25. Hunter KE, Seidler AL, Askie LM. Prospective registration trends, reasons for retrospective registration and mechanisms to increase prospective registration compliance: descriptive analysis and survey. *BMJ Open* 2018;8(3):e019983. doi: 10.1136/bmjopen-2017-019983
26. Dal-Re R, Ioannidis JP, Bracken MB, et al. Making prospective registration of observational research a reality. *Sci Transl Med* 2014;6(224):224cm1. doi: 10.1126/scitranslmed.3007513
27. Denneny C, Bourne S, Kolstoe SE. Registration audit of clinical trials given a favourable opinion by UK research ethics committees. *BMJ Open* 2019;9(2):e026840. doi: 10.1136/bmjopen-2018-026840
28. Ross JS, Mulvey GK, Hines EM, et al. Trial publication after registration in ClinicalTrials.Gov: a cross-sectional analysis. *PLoS Med* 2009;6(9):e1000144. doi: 10.1371/journal.pmed.1000144
29. Sun LW, Lee DJ, Collins JA, et al. Assessment of Consistency Between Peer-Reviewed Publications and Clinical Trial Registries. *JAMA Ophthalmol* 2019;137(5):552-56. doi: 10.1001/jamaophthalmol.2019.0312
30. Dickersin K. How important is publication bias? A synthesis of available data. *AIDS Educ Prev* 1997;9(1 Suppl):15-21.
31. Goldacre B, DeVito NJ, Heneghan C, et al. Compliance with requirement to report results on the EU Clinical Trials Register: cohort study and web resource. *BMJ* 2018;362:k3218. doi: 10.1136/bmj.k3218
32. European Medicines Agency. Posting of clinical trial summary results in European Clinical Trials Database (EudraCT) to become mandatory for sponsors as of 21 July 2014. [Available from: https://www.ema.europa.eu/en/news/posting-clinical-trial-summary-results-european-clinical-trials-database-eudract-become-mandatory; accessed 11/10/2019].
33. Food and Drug Administration Amendments Act of 2007. Pub L No 110-85, 121 Stat 823 (September 27, 2007).
34. Anderson ML, Chiswell K, Peterson ED, et al. Compliance with results reporting at ClinicalTrials.gov. *N Engl J Med* 2015;372(11):1031-9. doi: 10.1056/NEJMsa1409364
35. NIHR policy on clinical trial registration and disclosure of results. [Available from: https://www.nihr.ac.uk/documents/nihr-policy-on-clinical-trial-registration-and-disclosure-of-results/12252; accessed 11/10/2019].
36. Hopewell S, Loudon K, Clarke MJ, et al. Publication bias in clinical trials due to statistical significance or direction of trial results. *Cochrane Database Syst Rev* 2009(1):MR000006. doi: 10.1002/14651858.MR000006.pub3
37. Ioannidis JP. Effect of the statistical significance of results on the time to completion and publication of randomized efficacy trials. *JAMA* 1998;279(4):281-6. doi: 10.1001/jama.279.4.281
38. Petty S, Gross RA. Neurology(R) null hypothesis: A special supplement for negative, inconclusive, or confirmatory studies. *Neurology* 2018;91(1):12-13. doi: 10.1212/WNL.0000000000005803
39. Kiley R, Peatfield T, Hansen J, et al. Data Sharing from Clinical Trials - A Research Funder's Perspective. *N Engl J Med* 2017;377(20):1990-92. doi: 10.1056/NEJMsb1708278

For peer review only - http://bmjopen.bmj.com/site/about/guidelines.xhtml

40. Tudur Smith C, Hopkins C, Sydes MR, et al. How should individual participant data (IPD) from publicly funded clinical trials be shared? *BMC Med* 2015;13:298. doi: 10.1186/s12916-015-0532-z
41. Ohmann C, Banzi R, Canham S, et al. Sharing and reuse of individual participant data from clinical trials: principles and recommendations. *BMJ Open* 2017;7(12):e018647. doi: 10.1136/bmjopen-2017-018647
42. Banzi R, Canham S, Kuchinke W, et al. Evaluation of repositories for sharing individual-participant data from clinical studies. *Trials* 2019;20(1):169. doi: 10.1186/s13063-019-3253-3
43. Keerie C, Tuck C, Milne G, et al. Data sharing in clinical trials - practical guidance on anonymising trial datasets. *Trials* 2018;19(1):25. doi: 10.1186/s13063-017-2382-9
44. Clinical Study Data Request. [Available from: https://clinicalstudydatarequest.com/; accessed 11/10/2019].
45. The YODA Project. [Available from: https://yoda.yale.edu/; accessed 11/10/2019].
46. UK Data Service: ReShare. [Available from: https://reshare.ukdataservice.ac.uk/; accessed 11/10/2019].
47. Vivli - Center for Global Clinical Research Data. A global clinical research data sharing platform 2019 [Available from: https://vivli.org/; accessed 08/06/2019].
48. Burns NS, Miller PW. Learning What We Didn't Know - The SPRINT Data Analysis Challenge. *N Engl J Med* 2017;376(23):2205-07. doi: 10.1056/NEJMp1705323
49. Bierer BE, Crosas M, Pierce HH. Data Authorship as an Incentive to Data Sharing. *N Engl J Med* 2017;376(17):1684-87. doi: 10.1056/NEJMsb1616595
50. Lo B, DeMets DL. Incentives for Clinical Trialists to Share Data. *N Engl J Med* 2016;375(12):1112-5. doi: 10.1056/NEJMp1608351
51. Powell-Smith A, Goldacre B. The TrialsTracker: Automated ongoing monitoring of failure to share clinical trial results by all major companies and research institutions. *F1000Res* 2016;5:2629. doi: 10.12688/f1000research.10010.1
52. House of Commons Science and Technology Committee. (2018) 10th Report - Research integrity: clinical trials transparency. Written evidence submitted by Dr Simon Kolstoe (RES0030).

For peer review only

For peer review only - http://bmjopen.bmj.com/site/about/guidelines.xhtml

FIGURE LEGENDS

Figure 1: Flow Diagram

Figure 2: Percentage of trials registered in ISRCTN or other registry (total n=175 trials)

Figure 3: Period in months between award/trial end and reporting results (n=53 completed* trials)

Figure 4: Reporting (or publication) of main results by year when award ended

SUPPLEMENTARY FILES

Supplementary File 1: Policy timeline

Supplementary File 2: Search strategy

For peer review only

For peer review only - http://bmjopen.bmj.com/site/about/guidelines.xhtml

Figure 1 Flow Diagram

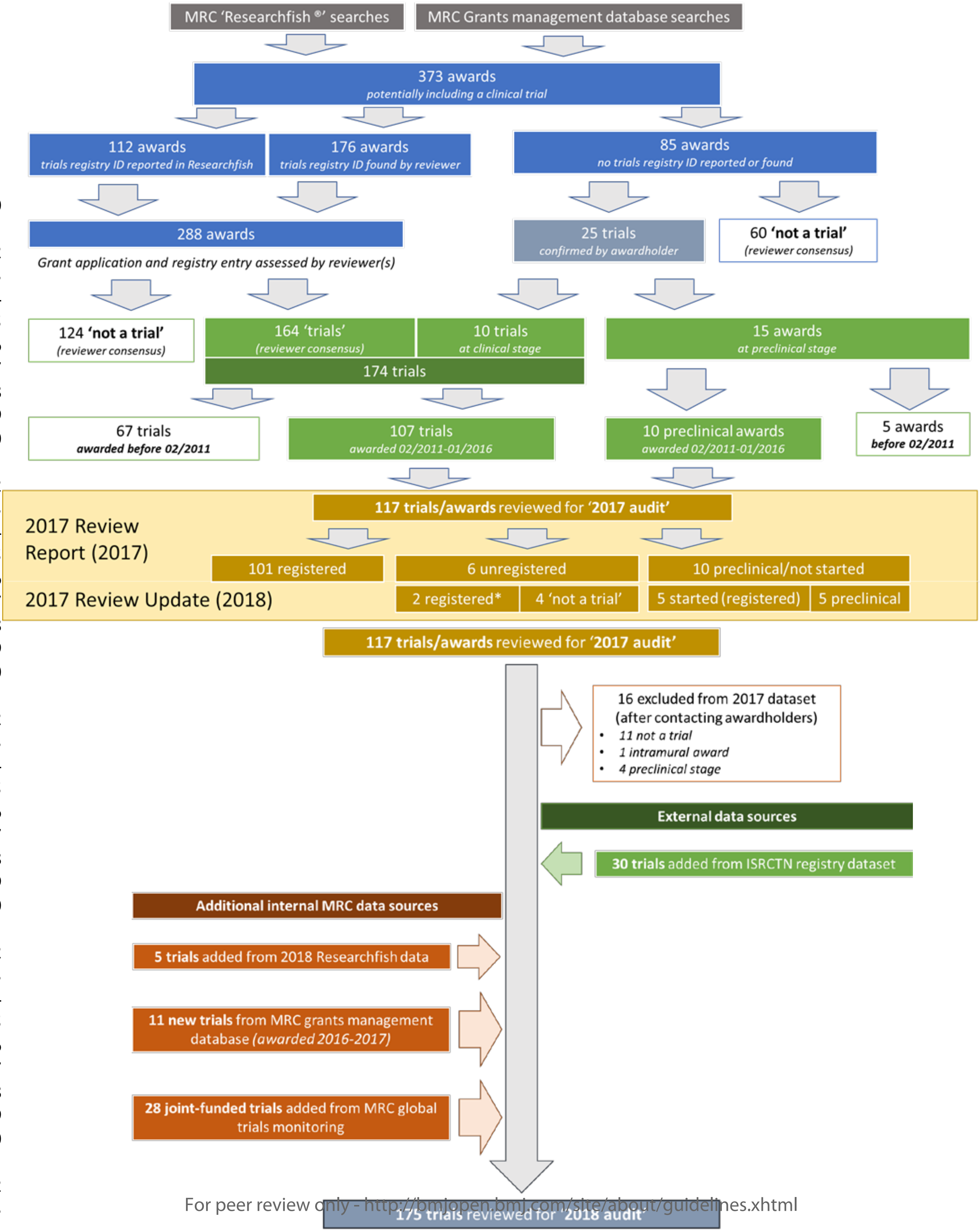

Figure 2 Percentage of trials registered in ISRCTN or other registry (total n=175 trials)

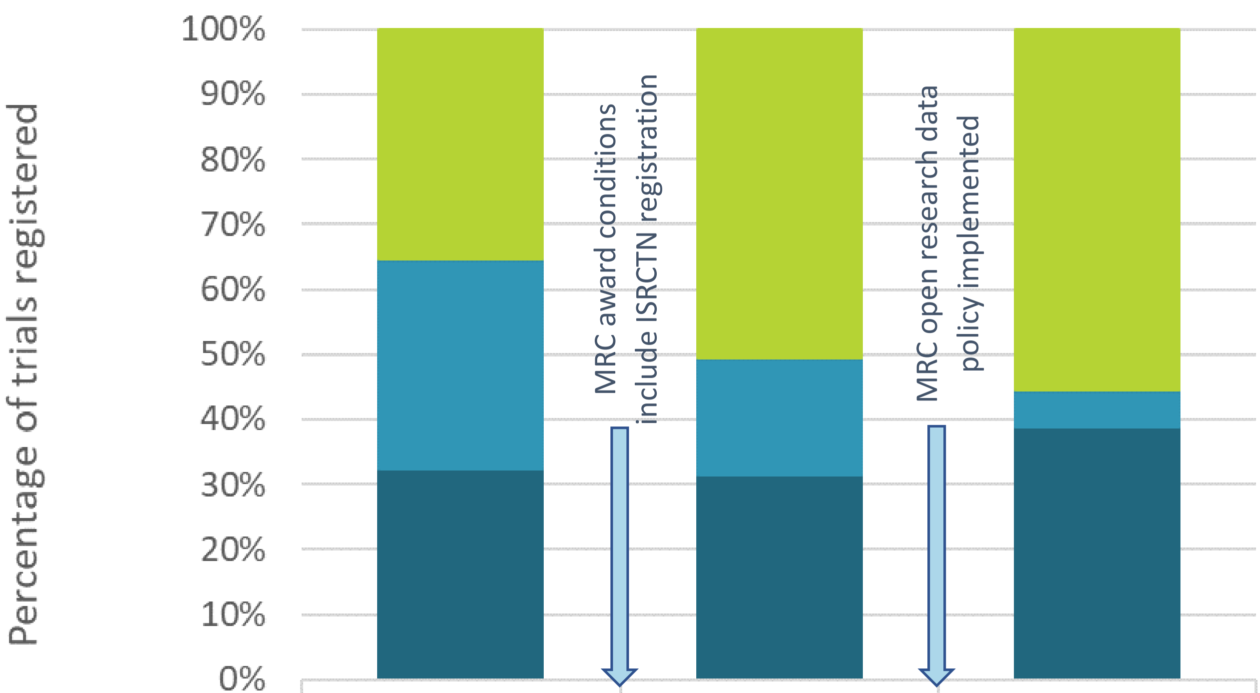

	2011-2013	2013-2016	2016-2017
Other Registry	24	45	10
ISRCTN (retrospective)	22	16	1
ISRCTN (prospective)	22	28	7

Note: All trials are counted once only. Trials registered in ISRCTN are counted as ISRCTN only; trials registered in any other registry (and not in ISRCTN) are counted once in 'Other' registries.

Figure 3 Period in months between award/trial end and reporting results (n=53 completed* trials)

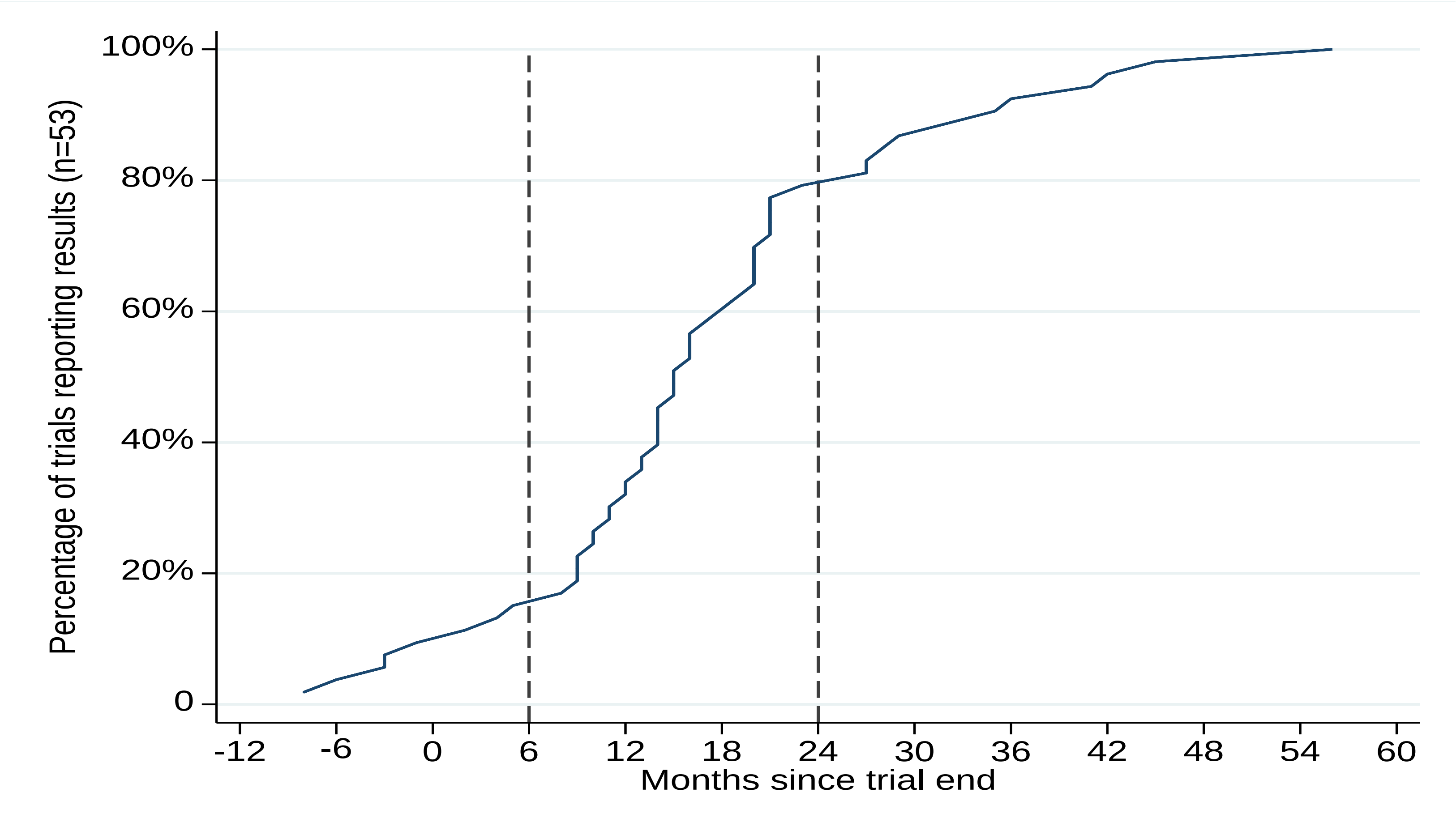

* Nine completed trials that have not yet reported findings were excluded

Figure 4 Reporting (or publication) of main results by year when award ended

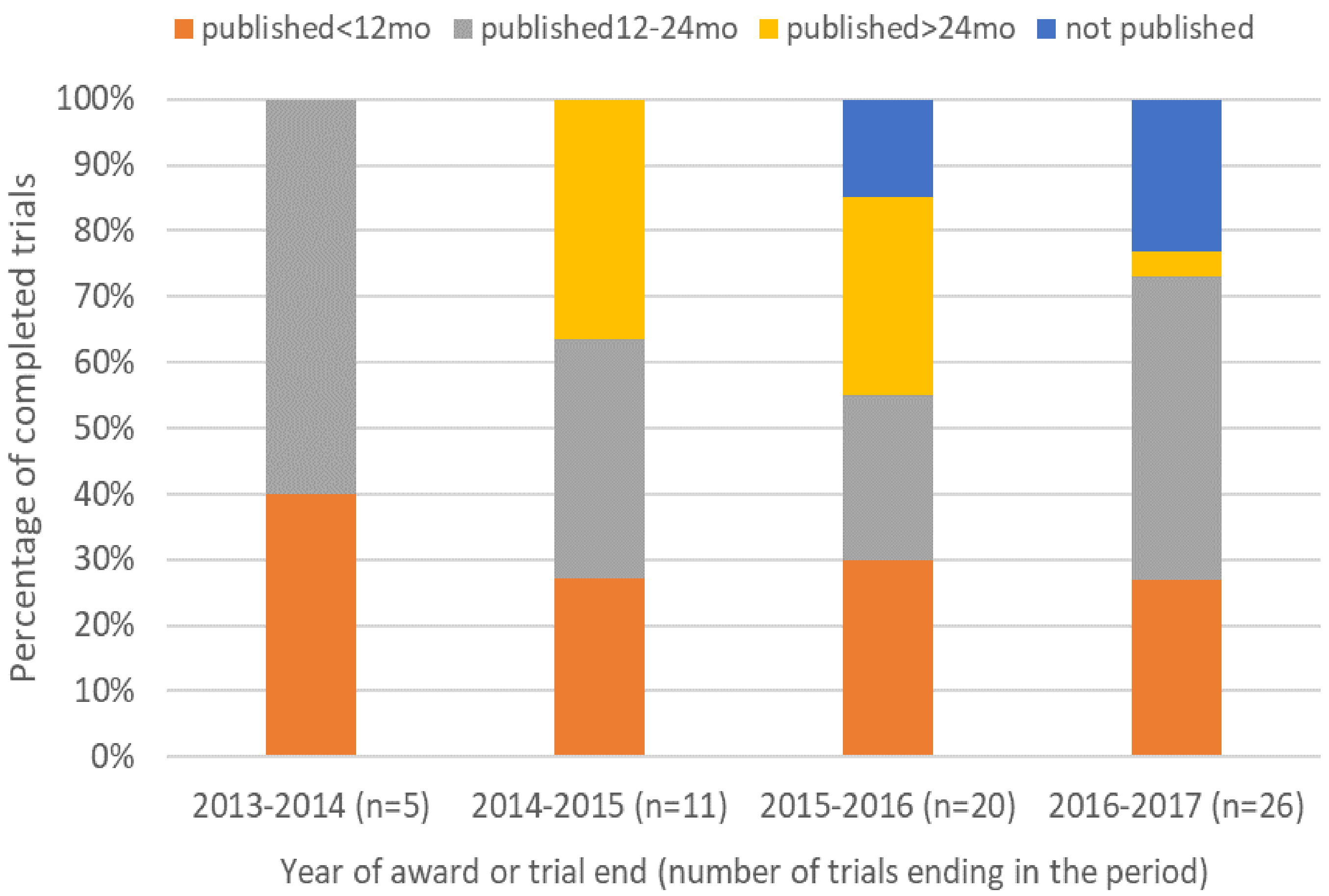

SUPPLEMENTARY FILE 1: POLICY TIMELINE

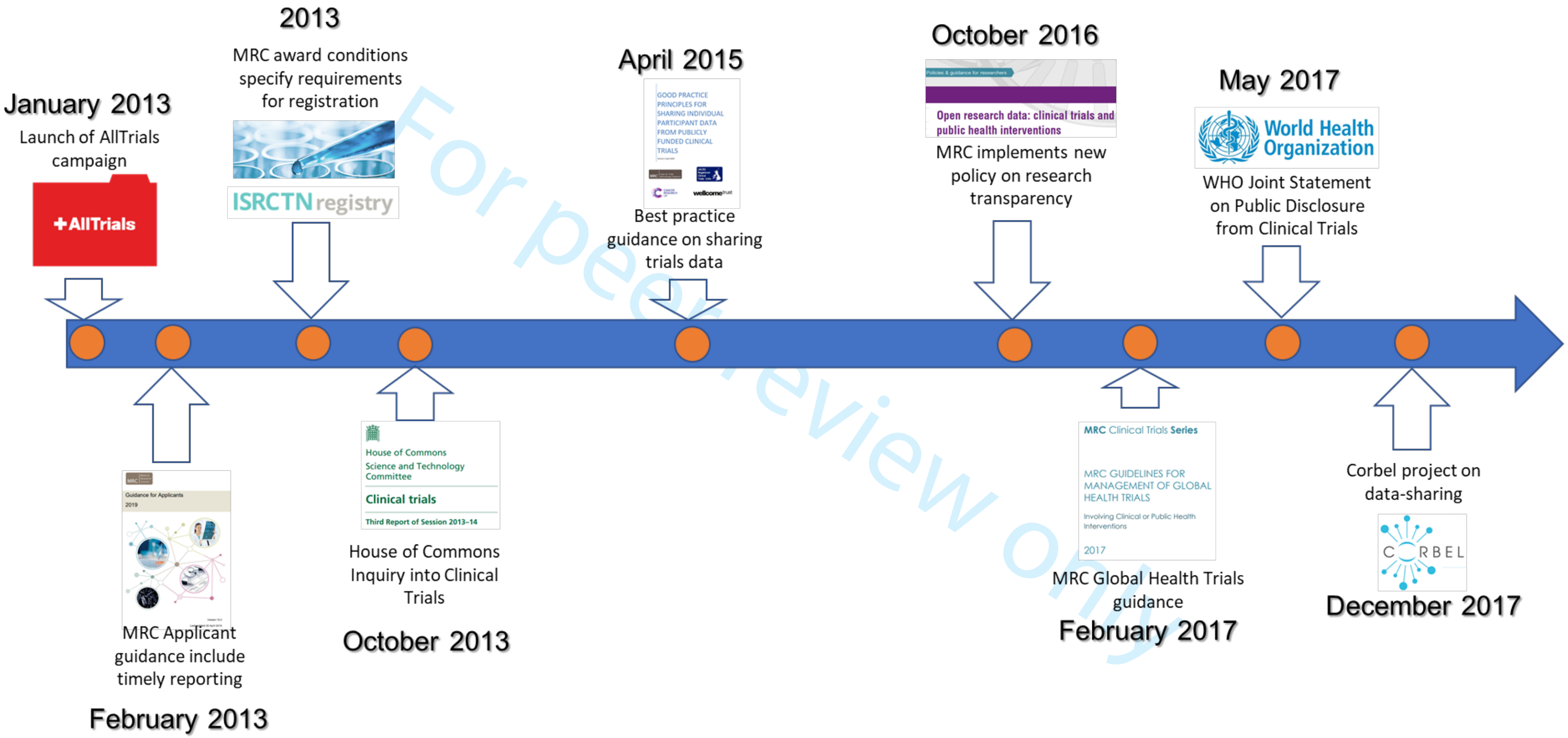

SUPPLEMENTARY FILE 2: SEARCH STRATEGY

Original Search Strategy:

1. Grant application/proposal classification includes any of the following keywords:
    a. 'MRC Trial'
    b. 'Clinical Trial'
    c. HRCS Research Activities AND Classification Name begins with [relevant classification code inserted]
2. Keyword search of 'Clinical Trial' on:
    a. Organisation or Department Name
    b. Joint Funder name
    c. Funding Scheme or Call
    d. Grant application:
        i. Technical Summary,
        ii. Lay Summary,
        iii. Impact Summary,
        iv. Aims and Objectives,
        v. Title,
        vi. Keywords.
3. Grant application submitted to:
    a. Board Science Late Phase Trials
    b. Board Science Methodology Research Panel

Concepts and Keywords used in the Revised Search Strategy (on all database fields):

1. Concept: Clinical Trials
    - Clinical Trials
    - Controlled Clinical Trials
    - Randomized Controlled Clinical Trials
    - Randomized Controlled Trials
    - Randomized Clinical Trials
2. Concept: Protocols
    - Clinical Protocols
    - Clinical Research Protocols
    - Treatment Protocols
3. Concept: Trials Monitoring
    - Clinical Trials Data Monitoring Committees
    - Data Monitoring Committees
    - Data and Safety Monitoring Boards
    - Safety Monitoring Boards
    - Phase 1 Clinical Trials
4. Concept: Phase I Trials
    - Phase I Clinical Trials
    - Phase 1 Clinical Trials
    - FDA Phase I Evaluation Studies
    - FDA Phase 1 Evaluation Studies
    - Human Microdosing Trials
    - FDA Phase 1 Drug Evaluation
    - FDA Phase I Drug Evaluation
5. Concept: Phase II Trials

For peer review only

- Phase II Clinical Trials
- FDA Phase II Evaluation Studies
- FDA Phase 2 Evaluation Studies
- FDA Phase II Drug Evaluation
- FDA Phase 2 Drug Evaluation

6. Concept: Phase 3 Clinical Trials
    - Phase 3 Clinical Trials
    - Phase III Clinical Trials
    - FDA Phase III Evaluation Studies
    - FDA Phase 3 Evaluation Studies
    - FDA Phase III Drug Evaluation
    - FDA Phase 3 Drug Evaluation
7. Concept: Phase 4 Clinical Trials
    - Phase 4 Clinical Trials
    - Phase IV Clinical Trials
    - FDA Phase IV Drug Evaluation
    - FDA Phase 4 Evaluation Studies
    - FDA Phase IV Evaluation Studies
    - FDA Phase 4 Drug Evaluation
8. Concept: Cohort Studies
    - Cohort studies
    - Closed Cohort Studies
    - Incidence Studies
    - Cohort Analysis
    - Historical Cohort Studies
    - Concurrent Studies
9. Concept: Control Groups
    - Control Groups
10. Concept: Crossover Design
    - Crossover Design
    - Cross-Over Trials
    - Crossover Trials
    - Cross-Over Design
    - Crossover Studies
11. Concept: Double-Blind Method
    - Double-Blind Method
    - Double-Masked Method
    - Double-Blind Study
    - Double-Masked Study
12. Concept: Feasibility Studies
    - Feasibility Studies
13. Concept: Informed Consent
    - Informed Consent
14. Concept: Intervention Studies
    - Intervention Studies
15. Concept: Mass Screening
    - Mass Screening
16. Concept: Multicenter Studies
    - Multicenter Studies
    - Multicenter Trials

For peer review only

17. Concept: Patient Compliance
    - Patient Compliance
    - Patient Non-Adherence
    - Patient Non-Compliance
    - Patient Nonadherence
    - Patient Cooperation
    - Patient Adherence
    - Patient Noncompliance
18. Concept: Placebos
    - Placebos
    - Sham Treatment
19. Concept: Research Design
    - Data Reporting
    - Research Technics
    - Data Adjustment
    - Research Proposal
    - Data Quality
    - Matched Groups
    - Research Techniques
    - Experimental Designs
    - Problem Formulation
    - Research Methodology
    - Scoring Methods
    - Error Sources
20. Concept: Single-Blind Method
    - Single-Blind Study
    - Single-Masked Method
    - Single-Masked Study
21. Concept: Investigational Therapies
    - Innovative Therapies
    - Experimental Therapies
    - Investigational Therapies
    - Investigational Treatments

For peer review only

Challenges for funders in monitoring compliance with policies on clinical trials registration and reporting: analysis of funding and registry data in the United Kingdom.

Journal:	*BMJ Open*
Manuscript ID	bmjopen-2019-035283.R1
Article Type:	Original research
Date Submitted by the Author:	09-Dec-2019
Complete List of Authors:	Knowles, Rachel; Medical Research Council<br>Ha, Kam Pou; Medical Research Council<br>Mueller, Julia ; Manchester University, Population Health, Health Services Research and Primary Care<br>Rawle, Frances; Medical Research Council<br>Parker, Rosa; Medical Research Council
**Primary Subject Heading**:	Health services research
Secondary Subject Heading:	Health policy
Keywords:	Clinical trials < THERAPEUTICS, STATISTICS & RESEARCH METHODS, AUDIT

SCHOLARONE™
Manuscripts

BMJ

I, the Submitting Author has the right to grant and does grant on behalf of all authors of the Work (as defined in the below author licence), an exclusive licence and/or a non-exclusive licence for contributions from authors who are: i) UK Crown employees; ii) where BMJ has agreed a CC-BY licence shall apply, and/or iii) in accordance with the terms applicable for US Federal Government officers or employees acting as part of their official duties; on a worldwide, perpetual, irrevocable, royalty-free basis to BMJ Publishing Group Ltd ("BMJ") its licensees and where the relevant Journal is co-owned by BMJ to the co-owners of the Journal, to publish the Work in this journal and any other BMJ products and to exploit all rights, as set out in our licence.

The Submitting Author accepts and understands that any supply made under these terms is made by BMJ to the Submitting Author unless you are acting as an employee on behalf of your employer or a postgraduate student of an affiliated institution which is paying any applicable article publishing charge ("APC") for Open Access articles. Where the Submitting Author wishes to make the Work available on an Open Access basis (and intends to pay the relevant APC), the terms of reuse of such Open Access shall be governed by a Creative Commons licence – details of these licences and which Creative Commons licence will apply to this Work are set out in our licence referred to above.

Other than as permitted in any relevant BMJ Author's Self Archiving Policies, I confirm this Work has not been accepted for publication elsewhere, is not being considered for publication elsewhere and does not duplicate material already published. I confirm all authors consent to publication of this Work and authorise the granting of this licence.

For peer review only

TITLE PAGE

Title: Challenges for funders in monitoring compliance with policies on clinical trials registration and reporting: analysis of funding and registry data in the United Kingdom.

Short title: Challenges for funders in monitoring compliance with policies on clinical trials registration and reporting.

Authors: Rachel L Knowles, Kam Pou Ha, Julia Mueller, Frances Rawle, Rosa Parker

Corresponding author:

Dr Rachel L Knowles

Medical Research Council (UK Research and Innovation)

58 Victoria Embankment

London EC4Y 0DS

United Kingdom

Email: rachel.knowles@mrc.ukri.org

Telephone: +44 (0)20 7395 2256

ORCiD: 0000-0002-5490-7682

Author affiliations:

Rachel L Knowles, Medical Research Council (UK Research and Innovation), London, United Kingdom

Kam Pou Ha, Medical Research Council (UK Research and Innovation), London, United Kingdom

Julia Mueller, Medical Research Council (UK Research and Innovation), London, United Kingdom (ORCiD: 0000-0002-4939-7112)

Frances Rawle, Medical Research Council (UK Research and Innovation), London, United Kingdom

Rosa Parker, Medical Research Council (UK Research and Innovation), London, United Kingdom

Word count: 4425 words (main text); abstract 274 words; 52 references; 4 figures; 2 tables

Keywords

clinical trials; registration of trials; disclosure of results; publication bias; outcome reporting; transparency; open science

For peer review only

For peer review only - http://bmjopen.bmj.com/site/about/guidelines.xhtml

ABSTRACT

Objectives:

To evaluate compliance by researchers with funder requirements on clinical trials transparency, including identifying key areas for improvement; to assess the completeness, accuracy and suitability for annual compliance monitoring of the data routinely collected by a research funding body.

Design:

Descriptive analysis of clinical trials funded between February 2011 and January 2017 against funder policy requirements.

Setting:

Public medical research funding body in the United Kingdom (UK).

Data sources:

Relevant clinical trials were identified from grant application details, post-award grant monitoring systems and the ISRCTN registry.

Main outcome measure:

Proportion of all Medical Research Council (MRC) funded clinical trials that were (a) registered in a clinical trials registry and (b) publicly reported summary results within 2 years of completion.

Results:

There were 175 grants awarded that included a clinical trial and all trials were registered in a public trials registry. Of 62 trials completed for over 24 months, 42 (68%) had publicly reported the main findings by 24 months after trial completion; 18 of these achieved this within 12 months of completion. 11 (18%) trials took longer than 24 months to report and 9 (15%) completed trials had not yet reported findings. Five datasets were shared with other researchers.

Conclusions:

Compliance with the funder policy requirements on trials registration was excellent. Reporting of the main findings was achieved for most trials within 24 months of completion, however the number of unreported trials remains a concern and should be a focus for future funder policy initiatives. Identifying trials from grant management and grant monitoring systems was challenging therefore funders should ensure investigators reliably provide trial registries with information and regularly update entries with details of trial publications and protocols.

For peer review only

For peer review only - http://bmjopen.bmj.com/site/about/guidelines.xhtml

ARTICLE SUMMARY

Strengths and limitations of this study

- The study evaluated the contribution made by different data sources to constructing a comprehensive dataset that allowed audit of grant holders compliance with one funder's clinical trials transparency policy.
- Information in the funder's databases for each trial was compared with the public registry entry for accuracy.
- Using grant applications, annual grant monitoring and trials registry data, the proportion of all funded clinical trials that were (i) publicly registered and (ii) for which results were publicly reported was calculated for a six-year period.
- Study strengths included the completeness of the dataset and careful validation of registration and publication details.
- Analyses were limited by the small number of trials per year.

For peer review only

INTRODUCTION

It is estimated that around 1 million clinical trials have been carried out since 1948, when the UK Medical Research Council (MRC) conducted a landmark randomised controlled trial on streptomycin in pulmonary tuberculosis.[1] Although responsibility for funding Phase 3 clinical trials in the UK transferred to the National Institute for Health Research (NIHR) in 2006, the MRC, as part of UK Research and Innovation, continues to directly fund early phase clinical trials, Phase 3 global health trials, other clinical and public health intervention studies, and to provide underpinning funding for trials methodology research and clinical trials units.

Serious concerns have been raised about the failure by researchers to report findings from a high proportion of clinical trials, as well as the selective reporting of 'positive' findings.[2-6] This represents not only a loss of valuable research findings but may lead to potential overestimation of the benefits of new treatments, with the risk that ineffective or harmful drugs or interventions are implemented in clinical practice. [3 7] Public trials registers, which facilitate the prospective registration of clinical trials, play a key role in reducing selective outcome reporting and publication bias[8] and the International Committee of Medical Journal Editors (ICMJE) requires prospective registration of trials as a condition of publication in its member journals.[9] The World Health Organization (WHO) has established an International Clinical Trials Registry Platform (ICTRP), which collects information on ongoing and completed trials from a network of primary registries across the world and provides a single point of public access.[10 11]

The AllTrials[12] campaign was established to actively promote clinical trials transparency *"All trials registered All trials reported"*, and funder signatories to the AllTrials petition, such as the UK MRC, subsequently strengthened requirements for grant recipients to prospectively register clinical trials and publicly disseminate results (Supplementary File 1). Despite this, a UK House of Commons Science and Technology Committee (STC) Inquiry into Research Integrity: Clinical Trials Transparency in 2018 concluded that *"registration is not yet universal … and reported outcomes do not always align with the original study proposal".* The Committee strongly recommended that compliance with reporting requirements should be monitored by funders and regulators and published at individual trial level.[13] This call for improved monitoring of compliance with registration and reporting policies echoed the WHO *Joint statement on public disclosure of results from clinical trials* to which 21 funding agencies have now signed up.[14]

As a founder signatory to the WHO joint statement, the MRC committed to developing ongoing monitoring of compliance with its *Policy on Open Research Data from Clinical Trials and Public Health Intervention Studies* published in October 2016[15] (Table 1). An initial review of research grants awarded and active over the five-year period 01 February 2011 to 31 January 2016 identified 107 clinical trials of which 101 (94%) were registered in a public trials registry. From 40 completed trials, 33 (82%) reported at least one publication.[16]

In this paper, we present a subsequent evaluation undertaken in 2018 that included MRC funding for awards involving a clinical trial up to 31 January 2017. Our aims were (1) to evaluate compliance by Principal Investigators (PIs; lead researcher or Grant Holder) with MRC's clinical transparency requirements as set out in the *Policy on Open Research Data from Clinical Trials and Public Health Intervention Studies* published in 2016[15] and to identify key areas for improvement, and (2) to assess data completeness and accuracy as a basis for the further development of annual compliance monitoring.

For peer review only - http://bmjopen.bmj.com/site/about/guidelines.xhtml

Table 1: Summary of MRC policy requirements (October 2016)*

**Registration**
1. Prospective registration of every MRC-funded clinical trial in the ISRCTN registry* prior to recruitment of first participant; *(*other WHO primary registries accepted if prior agreement with the MRC)*
2. Provision of the registry number to the MRC within 12 months of registration;
3. Reviewing (and updating if appropriate) the registry entry at least once per year until the trial has reported main results;
**Reporting**
4. Public report of the trial protocol – there should be a link to this from the registry entry (the protocol may be added as a supplementary document in the registry entry);
5. Public report of the trial's main results in a timely manner (usually within 12 months); there should be a link to the open accessible report/publication from the registry entry (and/or a report may be added as a supplementary document in the registry entry);
**Data Sharing**
1. Preparation of the trial dataset for sharing with external researchers is encouraged; researchers are expected to provide details of any datasets/databases created as part of an award in their annual report to the MRC.

* This table summarises requirements from the MRC policy[15]

METHODS

Data sources

The initial step was to compile a single monitoring dataset that included all awards made by the MRC during the audit period from 01 February 2011 until 31 January 2017, which included a clinical trial meeting the WHO definition: *"any research study that prospectively assigns human participants or groups of humans to one or more health-related interventions to evaluate the effects on health outcomes."*[17] The dataset included research grants, fellowship awards, global health trials, and any developmental clinical study award in which preclinical work was followed by a clinical trial. Trials funded through MRC Research Unit and MRC/University Unit programmes to Clinical Trials Units or joint-funding not directly managed by the MRC, for example the NIHR Efficiency and Mechanism Evaluation (EME) programme, were excluded. Information was extracted from four sources (Table 2): the MRC grants application and award database; the annual output, outcome and reporting system (Researchfish®); a listing of joint-funded global health trials for which the MRC had oversight; and an external dataset provided by the ISRCTN registry.[18]

Search methods (2017)

Basic keyword searches (Supplementary File 2) of MRC grants application and annual monitoring databases for the 2017 audit yielded 373 funded awards that potentially included a trial (Figure 1), and for 112 of these a trial registry number had been reported to the MRC. To complete missing entries, the grant reference, study title, acronym and PI name were entered into the three clinical trials registries most commonly used for UK research, the ISRCTN registry[18], ClinicalTrials.gov[19] and

For peer review only - http://bmjopen.bmj.com/site/about/guidelines.xhtml

European Union Clinical Trials Registry (EUCTR)[20], followed by an online web search using these terms.

Registered (n=124) and unregistered studies that did not meet the WHO clinical trial definition were excluded, as were registered trials (n=67) which began before the audit period. PIs of 25 unregistered but potential trials were contacted for further information; registry numbers were provided for nine trials and 15 were reported to be preclinical. One trial was unregistered but the research team proceeded to register it retrospectively. In total, 117 trials were included in a preliminary review in 2017.

Additional sources and updated searches (2018)

Following the collection of annual researcher reports in February 2018, the MRC 2017 dataset was updated and 16 studies were excluded (Figure 1). Eleven new awards made between 01 February 2016 and 31 January 2017 were added, as well as five studies that had progressed from preclinical research to a clinical trial during this period. Global health trials undergo additional governance monitoring by the MRC and this list yielded 28 additional trials not previously included.

A search of the independent ISRCTN registry dataset yielded 132 entries for trials starting on or after 01 February 2011 with the MRC named as the funder or sponsor. After review, 90 trials were not eligible (funder/sponsor was incorrect), 12 trials were already included, and 30 previously unidentified trials were added to the review dataset.

Confirming eligibility

175 awards made by the MRC between 01 February 2011 and 31 January 2017 were identified as including a clinical trial and eligible for inclusion in the 2018 review dataset.

Prior to commencing the review, project titles and abstracts of all awards were reviewed to confirm that they included a clinical trial. They were classified as a *trial*, *not a trial*, *trial not started* (preclinical) by two reviewers. PIs were contacted if uncertainty remained about whether the award included a clinical trial, or if registry numbers or main results publications could not be identified.

Evaluating the monitoring process: data completeness and accuracy

Each source contributing to the review dataset was individually assessed to determine the proportion of the total dataset that was ascertained.

To assess accuracy of the review dataset, specific data items were compared between the MRC award information and the registry entry for each trial; this included checking MRC grant reference numbers, award/trial dates, PI name and research organisation, and publications. Information from this comparison, as well as reviewing how search parameters such as funder name were recorded, was explored for trials that were and were not identified by each source to determine the reasons why differences occurred.

Evaluating researcher compliance with policy requirements

Analyses of compliance with the MRC *Policy on Open Research Data from Clinical Trials and Public Health Intervention Studies* (published October 2016) included the number and percentage of:

1. *all awards* that were registered in any public trials registry, and the percentage prospectively registered in the ISRCTN (as required by the policy);
2. *all registry entries* that had a link to the study protocol or publication/report;
3. *all completed awards* that had publicly reported main, or summary, results;
4. *all awards* that reported creating a dataset for sharing beyond the study team/collaborators.

For peer review only - http://bmjopen.bmj.com/site/about/guidelines.xhtml

Table 2 Sources of data for monitoring

Database	Purpose of database	Data entered by:	Data collected (relevant to monitoring)	Search strategy
**MRC Grants Application and Award database**	MRC application management and monitoring. Data is publicly available in UK Research and Innovation (UKRI) Gateway to Research.[21]	Applicants	Data entered as part of funding application and award process.<br>1. Award start and end dates<br>2. PI contact details<br>3. Study title and summary<br>4. Amount of award	**Automated** keyword search in title, abstract, study summary;
**MRC annual monitoring of awards (Researchfish®)**	MRC monitoring of activity and outputs from research funding. Data collected via the Researchfish® platform. Data is publicly available in UKRI Gateway to Research.[21]	Named PI receiving funding	Annual report/update to the MRC on 'live' or recently ended awards every February. Includes:<br>1. Clinical trial 'flag', i.e. trial associated with award<br>2. Trials registry number<br>3. Publications and other outputs<br>4. Datasets created/shared	**Automated** keyword search in title, abstract, study summary, outputs, publication fields; **Automated** search for clinical trials 'flag', entry for registry number
**MRC governance monitoring of global health trials**	MRC governance of global health clinical trials. Data not publicly available.	MRC staff	Information entered for governance and oversight<br>1. Record of Trial Steering Committee TSC dates<br>2. Changes to trial start and end dates	**Manual** search of study summary or notes to confirm study design and identify trials;
**ISRCTN registry[18]**	Publicly accessible and searchable database of individual clinical trials. Trial information is regularly submitted to WHO ICTRP portal where it is publicly available.[22]	Research team	Data entered at registration then updated or amended by research team as required.<br>1. Trial start and end dates<br>2. Contact person<br>3. Registration date<br>4. Links to protocol and main results<br>5. Trial funder<br>6. Trial sponsor<br>7. Date entry last updated	**Automated** search for Medical Research Council or MRC in funder and sponsor fields;

For peer review only - http://bmjopen.bmj.com/site/about/guidelines.xhtml

Changes in registration and reporting over time were conducted to investigate the impact of the MRC implementing the new policy that included *prospective* registration in 2016.[15] However the audit period also covered the earlier introduction into award terms and conditions, in 2013, of the requirement for trials to be registered, either retrospectively or prospectively.[23] Evaluation of the timing of registration relative to trial start was restricted to the ISRCTN registry as this was the only registry that reported whether registration was prospective or retrospective.

As information on award outputs is submitted annually by PIs using the Researchfish® system, there can be a delay in reporting of registration or publications of up to 12 months. Therefore, analysis of registration was restricted to awards in which the trial had started at least 12 months prior to the analysis data extract being taken (01 February 2018) and analysis of publications was restricted to studies in which the funding award or trial end date (the later of these dates) was at least 12 months before the data extract (referred to as *completed awards*).

Research publications or reports were only considered to report the main results from the trial if they (i) were defined in the ISRTCN registry entry as including the main results, (ii) included information in the publication stating that they reported the main results, or (iii) were confirmed by the PI to report the main results. The funding *award* end date was considered to be equivalent to the trial end date; however if the record in the registry stated that the *trial* end date was *after* the funding award end date, trials were considered completed on the *trial* end date. We refer throughout the paper to 'trial/award end' to capture this combined end point. Time to publication was therefore calculated using the *award* end date for most trials but using the *trial* end date reported in the registry when this was later than the award end date.

RESULTS

Completeness of trials data from different sources

The contribution that each of the four data sources (Table 2) made to the final review dataset was assessed. The original search strategy (Supplementary File 2) applied keywords to specific fields in the MRC grants application and annual monitoring databases but identified only 117 (67%) of 175 awards. Revised keyword searches (Supplementary File 2) were developed. These identified 913 studies, which were categorised by likelihood of including a clinical trial: category 1=highly likely to include a clinical trial [n=332]; category 2=a clinical intervention study or trial [n=274]; category 3=patient or cohort study [n=252]; category 4=clinical trial methods [n=32]; category 5=intervention study (e.g. community interventions, economic evaluations of interventions and other non-randomised interventions) [n=23]). The revised searches had higher sensitivity than the original search, identifying 149 (85% of 175) awards in the final review dataset, however they had poor positive predictive value as only 20% of retrieved awards included a clinical trial.

Assessment of annual reports to the MRC found that the trials registry number was not consistently reported and only 54 (29% of 175) awards were identified as including a trial via this data.

The 28 trials identified from global trials governance monitoring were not identified by the original keyword searches of MRC databases. They were not identified through the ISRCTN registry search, mainly because they received partnership funding and the MRC was not managing the grant.

The ISRCTN registry yielded 30 (17% of 175) trials that would otherwise have been missed. However, a further 90 (68% of the 132) studies identified by the ISRCTN search were excluded as checks showed that the study was either not a clinical trial or the trial did not receive MRC funding (although members of the study team may have received MRC funding for another research activity).

For peer review only - http://bmjopen.bmj.com/site/about/guidelines.xhtml

Therefore, both the search strategy and the accuracy of the registry entries presented significant challenges for accurate identification of relevant trials.

Accuracy of publicly available trials data

Review of study abstracts, or full applications, enabled exclusion of most studies that did not include a clinical trial. However it was necessary to contact PIs about 55 studies; 16 of these were subsequently excluded (Figure 1).

Information on MRC awards, including award reference, PI, research organisation and award dates are published in UK Research and Innovation (UKRI) Gateway to Research.[21] Trials registry entries report trial dates, the PI's name, a contact person (who may also be the PI), and the organisation acting as the trial sponsor. In 175 registry entries, the following information from UKRI Gateway to Research was reported: the research organisation name (n=159 [91%] of 175), PI name (n=114 [65%]), MRC award reference (n=60 [34%]) and the MRC named as funder (n=67 [38%]).

The dates of the trial in the registry entry matched the MRC award dates exactly in only five trials as most awards started before trial recruitment. The difference between these dates was even greater where awards included preclinical studies prior to commencement of the clinical trial.

Of 3420 journal articles reported by researchers in Researchfish® as outputs from the 175 MRC awards, only six were listed as the 'main results' publications in the ISRCTN registry.

Trials registration

All 175 (100%) trials were registered in a public trials registry; 96 (55%) of these in the ISRCTN registry and 79 (45%) with other registries, such as ClinicalTrials.gov and EUCTR. One-quarter (n=43) were registered in more than one registry. Registry entries for 84 (76%) of the 111 trials ongoing in March 2019 had been updated since 01 January 2017. The trial protocol, or an electronic link to the protocol, was provided in only 63 (36% of 175) registry entries.

The percentage of MRC trials registered prospectively in the ISRCTN registry increased over time: in 2011-2013 this was 22 (50.0%) of 44, in 2014-2015 it was 28 (63.6%) of 44 and in 2016-2017 it was 7 (87.5%) of 8 trials (Figure 2). In contrast, the introduction to award terms and conditions in 2013 of the requirement to use the ISRCTN registry exclusively and to acknowledge MRC funding in the registry entry, has yet to have a discernible impact.

Public reporting of main trial results

Sixty-two awards/trials were completed before 01 February 2017, of which 42 (68%) publicly reported main results, or posted results in a registry, within 24 months of award/trial end. Only 18 (29% of 62) trials reported within 12 months of award/trial end. A further 11 (18% of 62) completed trials reported main results more than 24 months after award/trial end. Nine trials had not reported results (at March 2019).

Of 62 completed awards, 40 (65%) had a link to the main results or posted results in the registry (links=40; results posted=4; both were added for some trials).

The time taken to report results, for the 53 completed and reported trials, ranged from 8 months prior to 56 months after award/trial end, and the majority reported between 6 and 24 months after award/trial end (Figure 3).

The PIs for the 9 unreported trials were contacted for further information; 7 reported that papers were progressing and publication was expected within the next 12 months. The period since trial

For peer review only - http://bmjopen.bmj.com/site/about/guidelines.xhtml

completion ranged from 24 to 43 months; various reasons for delay were highlighted, including absence of a key researcher, negative findings or early trial termination. One award had included findings in a press release and symposium abstract but these were insufficiently detailed to be considered main results.

There was an increase in the percentage of trials reporting results before 24 months in 2016-2017 compared with most previous years; in contrast, the percentage of trials that reported within 12 months changed little over time (Figure 4). In recent years, the percentage of unreported trials increased, reflecting the higher number of trials ending in these years as well as delays in reporting. Based on our experience between 2017 and 2018, it appears likely that the total number of unreported trials will reduce further with late publications.

Data-sharing

PIs reported creating 18 datasets for sharing with other researchers as an output from their clinical trial data, representing 10% of all 175 trials. Five of these datasets had been shared with researchers outside the original study team, one had been deposited in a repository for reuse by other researchers, and one was available through a disease registry.

DISCUSSION

As a signatory to AllTrials[12] and the WHO Joint Statement[14], the MRC has made a commitment to the implementation of regular monitoring of researchers' compliance with the registration and reporting requirements for clinical trials set out in its *Policy on Open Research Data from Clinical Trials and Public Health Intervention Studies (2016)*.[15] Although the review found that all 175 trials funded by the MRC between 2011 and 2017 were registered, only half were registered in the WHO primary registry specifically named in the MRC award conditions and researchers often provided different information in the registry entry to that held by the funder. Summary results were reported within two years of completion for almost 70% of trials; most through academic papers but two-thirds also added links to these results in the trials registry entry. Despite this the percentage of trials that had been completed for over two years but had failed to report was unacceptably high (15%). This underlines the need for renewed joint efforts by the MRC to address the barriers to timely publication that were identified, which included delays in publishing after early trial termination or if findings were negative, and research staff absence or turnover.

Registration

MRC-funded researchers are required to register trials in the ISRCTN registry because it permits registration of the wide range of study designs supported by the MRC and, as a WHO primary registry, contributes regularly to the ICTRP[11] maximising access to trials information. MRC policy explicitly states that researchers must add trial protocols and publications, as well as regularly reviewing and updating registry entries to ensure these remain accurate. An important positive finding was the decrease in the proportion of trials registering retrospectively, a trend that has also been noted by other authors and attributed to rising awareness among researchers of the need to register their trials.[24-26] However, our review is consistent with previous reports[27 28] that have highlighted inaccurate and incomplete registry entries as a significant cause for concern. This suggests that registries should review their guidance and controls around data entry by researchers to improve the consistency and accuracy of trial records. Disappointingly, a high proportion of trials in our review were not registered in the ISRCTN registry despite the MRC reimbursing the registration fee, and it was also noticeable that information in many registry entries was discrepant with that held by the MRC. Furthermore only one-third of registry entries included a link to the trial

For peer review only - http://bmjopen.bmj.com/site/about/guidelines.xhtml

protocol, despite early protocol publication being key to minimising publication bias and selective reporting of trial findings.[14 29] These findings suggest that researchers do not fully appreciate their responsibilities with regard to clinical trials transparency, specifically the need to regularly update registry entries so that they provide accurate and complete information to participants, the public and the research community. We also observed that the responsibility for complying with clinical trials transparency requirements falls largely upon the shoulders of individual researchers and would therefore strongly endorse the call by Goldacre et al[30] for trial sponsors and research organisations which host trials to take greater responsibility for promoting and monitoring transparency, providing more administrative support, and raising awareness and compliance among researchers.

Reporting results

Funder signatories to the WHO Joint Statement[14] committed to developing policies on timely publication, including an indicative timeframe of 24 months for publication in a journal and working towards a timeframe of 12 months for reporting summary results in a registry. Current MRC policy includes an expectation that main trial results will be reported "usually" within 12 months, but this is not mandated. European Union (EU) and United States (US) clinical trials legislation mandate the posting of trial results by 12 months after trial end[31 32] although this is often not achieved[30 33], while other non-commercial funders have mandated timeframes ranging from 12 to 24 months after trial completion.[7 34] Our review confirms 24 months as an achievable timeframe for publication in peer reviewed journals for MRC PIs; therefore including a policy requirement for all trials to *either* publish *or* report findings in the registry by 24 months would encourage significant improvement on current practice. We also demonstrated that contacting researchers about reporting was an effective prompt that resulted in submission for publication. This is consistent with the findings of the national audit undertaken by the UK Health Research Authority (HRA)[26] and could be easily integrated into the routine annual review process by funders, registries and regulators. Although trials with null or negative findings often experience longer delays to publication[35 36], there is increasing recognition by journals of the importance of null studies. This is underlined by the development of journals focusing solely on such studies, initiatives such as the recent special supplement of Neurology on the null hypothesis[37], as well as the introduction of the "registered reports" model to some journals.

Data sharing

Funders are increasingly encouraging researchers to make their data available after study completion to maximise the opportunity for health improvement from public investment in research[38], however we found little evidence of trial datasets being prepared or made available for reuse by researchers. In recent years, significant efforts have been made to develop guidance[39-42] and services to enable researchers to share data, including data repositories and online catalogues, independent access review committees, secure platforms for data access and analysis, and data anonymisation tools to reduce disclosure risk.[43-46] There have also been various initiatives to increase academic interest and innovation in data sharing, such as the SPRINT challenge.[47] However, as yet, these incentives to share data do not appear to be sufficiently persuasive and a central concern remains the failure to adequately recognise or 'credit' those who create important data resources for use by other researchers.[48 49]

Sustainable and effective compliance monitoring

Conducting this review demonstrated that deriving a complete trials dataset for annual monitoring currently requires the cross-validation of data extracts from multiple existing sources. No single

For peer review only - http://bmjopen.bmj.com/site/about/guidelines.xhtml

source contributed more than 85% of the final dataset, nor does any have sufficiently high levels of accuracy to be relied upon as the sole data source for compliance monitoring at present. Importantly PIs failed to report the registry number for their trial to the MRC for 71% of trials in this audit, indicating a concerning lack of compliance with this basic requirement.

Inaccuracy and incomplete data was often due to a failure by researchers to maintain and update registry entries as has previously been reported.[28 50] Although the ISRCTN registry does not provide a template for posting results, it was a reliable source of information about the publication of summary results as it actively followed-up with researchers to confirm the main results publications for completed studies. In contrast, although both EUCTR and ClinicalTrials.gov provide templates for posting results directly into the registry, only four of the studies in our dataset (1 in EUCTR and 3 in ClinicalTrials.gov) had completed these.

Although keyword searches successfully identified many trials, our review required significant commitment of staff time to perform extensive internet searches, check the accuracy of data, and cross-validate information from different sources. This included visual checks of registry entries and grant applications to ensure that studies were eligible trials and that information was complete; this was only possible with the help of Policy Interns undertaking placements as part of an MRC-funded PhD studentship. Importantly over 30% of PIs had to be contacted directly one or more times to confirm that a study had progressed beyond the preclinical phase, or to provide registry numbers or publication details. Although initiatives using automated monitoring to identify trials which have failed to report findings have been pursued elsewhere[30 50], it is not currently possible to 'automate' the compliance monitoring process for MRC awards. The challenge of introducing automated methods to monitor trial publications was also highlighted in the HRA national audit of ethical approvals.[26 51] Although it is not feasible for the MRC to commit such a high level of staff and resources to carrying out this manual verification process every year, the dataset created during the current review now reflects the complete clinical trials portfolio and can be supplemented each year with information about new funding awards. The key to further reducing the burden of compliance monitoring for funders and regulators is likely to be greater reliance on registry data, therefore it is essential to focus on strengthening requirements for researchers to maintain accurate, complete and regularly updated registry entries for all ongoing trials.

Strengths and limitations

As with the national audit undertaken by the UK Health Research Authority (HRA)[26], our review was based on a 'complete' denominator, comprising all MRC awards including a clinical trial during the review period, so accurately reflects registration and publication rates. PIs were contacted if there was any uncertainty about whether a study met the WHO definition of a clinical trial and studies that were not required to register under our policy were excluded, for example, non-interventional, genetic or biomarker studies, or studies that failed to progress beyond the preclinical stages.

A limitation of our review dataset was the relatively small number of awards, in particular completed trials, during the period, which had an impact on analyses of publication rates and investigation of trends. We did not attempt to extend the dataset before 2011 as the MRC did not introduce applicant guidance on trials registration until 2013 so this would not have reflected contemporary practice however, in future reviews, additional years of data will be added to the current dataset. We were also restricted by the information available, for example we could only explore prospective registration in the ISRCTN registry as the other registries did not report this, however we would expect the proportion prospectively registered to be similar across registries.

For peer review only - http://bmjopen.bmj.com/site/about/guidelines.xhtml

CONCLUSIONS

As the current compliance review mainly included MRC awards before the publication of the new transparency policy, it is perhaps best considered as a reflection of existing practice. However, it will provide a clear benchmark for assessing improvement in clinical trials registration and reporting in future years. This review highlighted some key issues for funders consideration when establishing compliance monitoring including (1) developing the use of registry data as a primary source for compliance monitoring, (2) strengthening funder requirements for registry entries to be maintained, (3) improving identification of funded awards that include trials to ensure a complete denominator for evaluating compliance with registration, and (4) including the research organisation, registry number and award reference number in compliance monitoring reports to allow further details of individual trials to be found in public registries.

As a funder of a complex clinical trials portfolio, spanning early phase translational clinical trials as well as late phase trials involving behavioural, psychological and public health interventions, the MRC has a keen interest in promoting research transparency across all research involving human participants regardless of study design. An initial step has been to extend the MRC *Policy on Open Research Data from Clinical Trials and Public Health Intervention Studies* (2016). We are now working towards the inclusion of explicit statements about transparency within the data management plans of all funded clinical and public health studies.

For peer review only

For peer review only - http://bmjopen.bmj.com/site/about/guidelines.xhtml

Acknowledgements: We would like to thank staff at the ISRCTN registry and within the MRC Evaluation Team for providing the data for the review.

Author contributions: RK**,** RP and FR conceived and designed the study. JM, KPH and RK extracted and analysed data and all authors interpreted the data. RK and RP drafted the manuscript; all authors reviewed and approved the final manuscript. RK is first author and guarantor. The corresponding author attests that all listed authors meet authorship criteria and that no others meeting the criteria have been omitted.

Competing interests: All authors have completed the ICMJE uniform competing interest form (available on request from the corresponding author).

Funding: JM and KPH were funded by MRC Policy Internships. This research received no specific grant from any funding agency in the public, commercial or not-for-profit sectors.

Ethical approval: Not required.

Data sharing: The dataset is available on the MRC website as a downloadable file or on enquiry to the MRC.

Transparency: The lead author (RK) affirms that the manuscript is an honest, accurate, and transparent account of the study being reported; that no important aspects of the study have been omitted; and that any discrepancies from the study as planned have been explained.

This is an Open Access article distributed in accordance with the Creative Commons Attribution Non Commercial (CC BY-NC 4.0) license, which permits others to distribute, remix, adapt, build upon this work non-commercially, and license their derivative works on different terms, provided the original work is properly cited and the use is non-commercial. See: http://creativecommons.org/licenses/by-nc/4.0/

Patient and Public Involvement: This research was done without patient or public involvement. Neither patients nor the public were invited to comment on the study design or results, or contribute to the writing of the paper. A lay language summary of the review will be placed on the MRC website to disseminate findings.

For peer review only

REFERENCES

1. Streptomycin treatment of pulmonary tuberculosis. *Br Med J* 1948;2(4582):769-82
2. Song F, Parekh S, Hooper L, et al. Dissemination and publication of research findings: an updated review of related biases. *Health Technol Assess* 2010;14(8):iii, ix-xi, 1-193. doi: 10.3310/hta14080
3. Chalmers I, Glasziou P. Avoidable waste in the production and reporting of research evidence. *Lancet* 2009;374(9683):86-9. doi: 10.1016/S0140-6736(09)60329-9bays1
4. Antes G, Chalmers I. Under-reporting of clinical trials is unethical. *Lancet* 2003;361(9362):978-9. doi: 10.1016/S0140-6736(03)12838-3
5. Dickersin K, Chan S, Chalmers TC, et al. Publication bias and clinical trials. *Control Clin Trials* 1987;8(4):343-53
6. Hahn S, Williamson PR, Hutton JL. Investigation of within-study selective reporting in clinical research: follow-up of applications submitted to a local research ethics committee. *J Eval Clin Pract* 2002;8(3):353-9
7. DeVito NJ, French L, Goldacre B. Noncommercial Funders' Policies on Trial Registration, Access to Summary Results, and Individual Patient Data Availability. *JAMA* 2018;319(16):1721-23. doi: 10.1001/jama.2018.2841
8. Dickersin K, Rennie D. Registering clinical trials. *JAMA* 2003;290(4):516-23. doi: 10.1001/jama.290.4.516
9. De Angelis C, Drazen JM, Frizelle FA, et al. Clinical trial registration: a statement from the International Committee of Medical Journal Editors. *Lancet* 2004;364(9438):911-2. doi: 10.1016/S0140-6736(04)17034-7
10. Gulmezoglu AM, Pang T, Horton R, et al. WHO facilitates international collaboration in setting standards for clinical trial registration. *Lancet* 2005;365(9474):1829-31. doi: 10.1016/S0140-6736(05)66589-0
11. World Health Organization. International Clinical Trials Registry Platform (ICTRP) - Primary Registries. [Available from: https://www.who.int/ictrp/network/primary/en/; accessed 11/10/2019]
12. AllTrials. All Trials Registered - All Trials Reported. [Available from: http://www.alltrials.net/find-out-more/; accessed 08/06/2019]
13. House of Commons Science and Technology Committee (2018). 10th report - Research integrity: clinical trials transparency. HC1480.
14. World Health Organization (2017). Joint statement on public disclosure of results from clinical trials. [Available from: https://www.who.int/ictrp/results/ICTRP_JointStatement_2017.pdf?ua=1; accessed 08/06/2019]
15. UK Medical Research Council (2016). Policy on Open Research Data from Clinical Trials and Public Health Intervention Studies (2016). [Available from: https://mrc.ukri.org/research/policies-and-guidance-for-researchers/open-research-data-clinical-trials-and-public-health-interventions/; accessed 11/10/2019]
16. House of Commons Science and Technology Committee (2018). 10th Report - Research integrity: clinical trials transparency. Written evidence submitted by the Medical Research Council (RES0041).
17. World Health Organization. Health topics: Clinical trials. [Available from: https://www.who.int/topics/clinical_trials/en/; accessed 11/10/2019]
18. ISRCTN Registry [Available from: https://www.isrctn.com/; accessed 11/10/2019]
19. ClinicalTrials.gov [Available from: https://clinicatrials.gov/; accessed 11/10/2019]
20. About the EU Clinical Trials Register [Available from: https://www.clinicaltrialsregister.eu/about.html; accessed 11/10/2019]
21. UK Research and Innovation Gateway to Research. [Available from: https://www.gtr.ukri.org/; accessed 11/10/2019]

For peer review only - http://bmjopen.bmj.com/site/about/guidelines.xhtml

22. World Health Organization. International Clinical Trials Registry Platform (ICTRP). [Available from: https://www.who.int/ictrp; accessed 08/06/2019]
23. House of Commons Science and Technology Committee (2013). Seventh Special Report. Clinical trials: Medical Research Council Response to the Committee's Third Report of Session 2013-14. Appendix 1.
24. Hunter KE, Seidler AL, Askie LM. Prospective registration trends, reasons for retrospective registration and mechanisms to increase prospective registration compliance: descriptive analysis and survey. *BMJ Open* 2018;8(3):e019983. doi: 10.1136/bmjopen-2017-019983
25. Dal-Re R, Ioannidis JP, Bracken MB, et al. Making prospective registration of observational research a reality. *Sci Transl Med* 2014;6(224):224cm1. doi: 10.1126/scitranslmed.3007513
26. Denneny C, Bourne S, Kolstoe SE. Registration audit of clinical trials given a favourable opinion by UK research ethics committees. *BMJ Open* 2019;9(2):e026840. doi: 10.1136/bmjopen-2018-026840
27. Ross JS, Mulvey GK, Hines EM, et al. Trial publication after registration in ClinicalTrials.Gov: a cross-sectional analysis. *PLoS Med* 2009;6(9):e1000144. doi: 10.1371/journal.pmed.1000144
28. Sun LW, Lee DJ, Collins JA, et al. Assessment of Consistency Between Peer-Reviewed Publications and Clinical Trial Registries. *JAMA Ophthalmol* 2019;137(5):552-56. doi: 10.1001/jamaophthalmol.2019.0312
29. Dickersin K. How important is publication bias? A synthesis of available data. *AIDS Educ Prev* 1997;9(1 Suppl):15-21.
30. Goldacre B, DeVito NJ, Heneghan C, et al. Compliance with requirement to report results on the EU Clinical Trials Register: cohort study and web resource. *BMJ* 2018;362:k3218. doi: 10.1136/bmj.k3218
31. European Medicines Agency. Posting of clinical trial summary results in European Clinical Trials Database (EudraCT) to become mandatory for sponsors as of 21 July 2014. [Available from: https://www.ema.europa.eu/en/news/posting-clinical-trial-summary-results-european-clinical-trials-database-eudract-become-mandatory; accessed 11/10/2019]
32. Food and Drug Administration Amendments Act of 2007. Pub L No 110-85, 121 Stat 823 (September 27, 2007)
33. Anderson ML, Chiswell K, Peterson ED, et al. Compliance with results reporting at ClinicalTrials.gov. *N Engl J Med* 2015;372(11):1031-9. doi: 10.1056/NEJMsa1409364
34. NIHR policy on clinical trial registration and disclosure of results. [Available from: https://www.nihr.ac.uk/documents/nihr-policy-on-clinical-trial-registration-and-disclosure-of-results/12252; accessed 11/10/2019]
35. Hopewell S, Loudon K, Clarke MJ, et al. Publication bias in clinical trials due to statistical significance or direction of trial results. *Cochrane Database Syst Rev* 2009(1):MR000006. doi: 10.1002/14651858.MR000006.pub3
36. Ioannidis JP. Effect of the statistical significance of results on the time to completion and publication of randomized efficacy trials. *JAMA* 1998;279(4):281-6. doi: 10.1001/jama.279.4.281
37. Petty S, Gross RA. Neurology(R) null hypothesis: A special supplement for negative, inconclusive, or confirmatory studies. *Neurology* 2018;91(1):12-13. doi: 10.1212/WNL.0000000000005803
38. Kiley R, Peatfield T, Hansen J, et al. Data Sharing from Clinical Trials - A Research Funder's Perspective. *N Engl J Med* 2017;377(20):1990-92. doi: 10.1056/NEJMsb1708278
39. Tudur Smith C, Hopkins C, Sydes MR, et al. How should individual participant data (IPD) from publicly funded clinical trials be shared? *BMC Med* 2015;13:298. doi: 10.1186/s12916-015-0532-z
40. Ohmann C, Banzi R, Canham S, et al. Sharing and reuse of individual participant data from clinical trials: principles and recommendations. *BMJ Open* 2017;7(12):e018647. doi: 10.1136/bmjopen-2017-018647

For peer review only

For peer review only - http://bmjopen.bmj.com/site/about/guidelines.xhtml

41. Banzi R, Canham S, Kuchinke W, et al. Evaluation of repositories for sharing individual-participant data from clinical studies. *Trials* 2019;20(1):169. doi: 10.1186/s13063-019-3253-3
42. Keerie C, Tuck C, Milne G, et al. Data sharing in clinical trials - practical guidance on anonymising trial datasets. *Trials* 2018;19(1):25. doi: 10.1186/s13063-017-2382-9
43. Clinical Study Data Request. [Available from: https://clinicalstudydatarequest.com/; accessed 11/10/2019]
44. The YODA Project. [Available from: https://yoda.yale.edu/; accessed 11/10/2019]
45. UK Data Service: ReShare. [Available from: https://reshare.ukdataservice.ac.uk/; accessed 11/10/2019]
46. Vivli - Center for Global Clinical Research Data. A global clinical research data sharing platform. [Available from: https://vivli.org/; accessed 08/06/2019]
47. Burns NS, Miller PW. Learning What We Didn't Know - The SPRINT Data Analysis Challenge. *N Engl J Med* 2017;376(23):2205-07. doi: 10.1056/NEJMp1705323
48. Bierer BE, Crosas M, Pierce HH. Data Authorship as an Incentive to Data Sharing. *N Engl J Med* 2017;376(17):1684-87. doi: 10.1056/NEJMsb1616595
49. Lo B, DeMets DL. Incentives for Clinical Trialists to Share Data. *N Engl J Med* 2016;375(12):1112-5. doi: 10.1056/NEJMp1608351
50. Powell-Smith A, Goldacre B. The TrialsTracker: Automated ongoing monitoring of failure to share clinical trial results by all major companies and research institutions. *F1000Res* 2016;5:2629. doi: 10.12688/f1000research.10010.1
51. House of Commons Science and Technology Committee (2018). 10th Report - Research integrity: clinical trials transparency. Written evidence submitted by Dr Simon Kolstoe (RES0030).

For peer review only

For peer review only - http://bmjopen.bmj.com/site/about/guidelines.xhtml

FIGURE LEGENDS

Figure 1: Flow Diagram

Figure 2: Percentage of trials registered in ISRCTN or other registry (total n=175 trials)

Figure 3: Period in months between award/trial end and reporting results (n=53 completed* trials)

Figure 4: Reporting (or publication) of main results by year when award ended

SUPPLEMENTARY FILES

Supplementary File 1: Policy timeline

Supplementary File 2: Search strategy

For peer review only

For peer review only - http://bmjopen.bmj.com/site/about/guidelines.xhtml

Figure 1 Flow Diagram

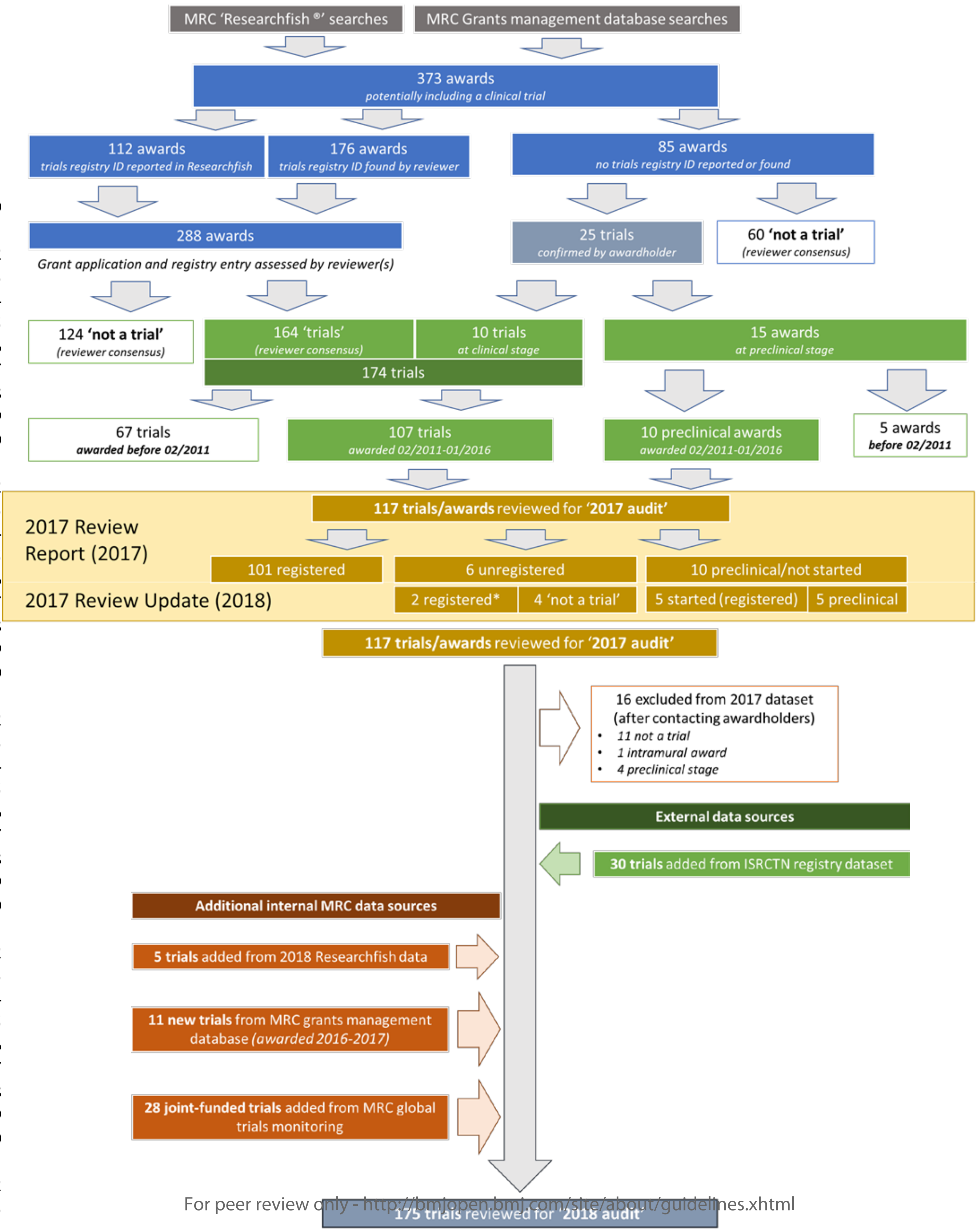

Figure 2 Percentage of trials registered in ISRCTN or other registry (total n=175 trials)

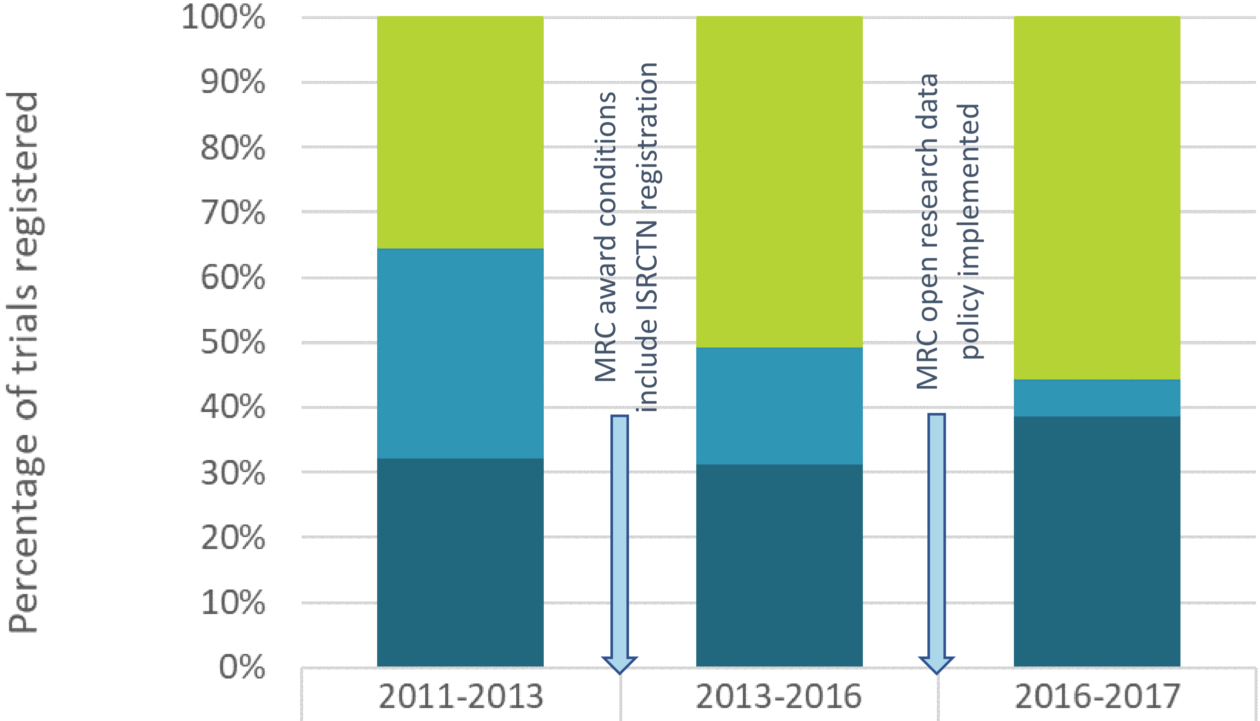

	2011-2013	2013-2016	2016-2017
Other Registry	24	45	10
ISRCTN (retrospective)	22	16	1
ISRCTN (prospective)	22	28	7

Note: All trials are counted once only. Trials registered in ISRCTN are counted as ISRCTN only; trials registered in any other registry (and not in ISRCTN) are counted once in ‘Other’ registries.

For peer review only - http://bmjopen.bmj.com/site/about/guidelines.xhtml

Figure 3 Period in months between award/trial end and reporting results (n=53 completed* trials)

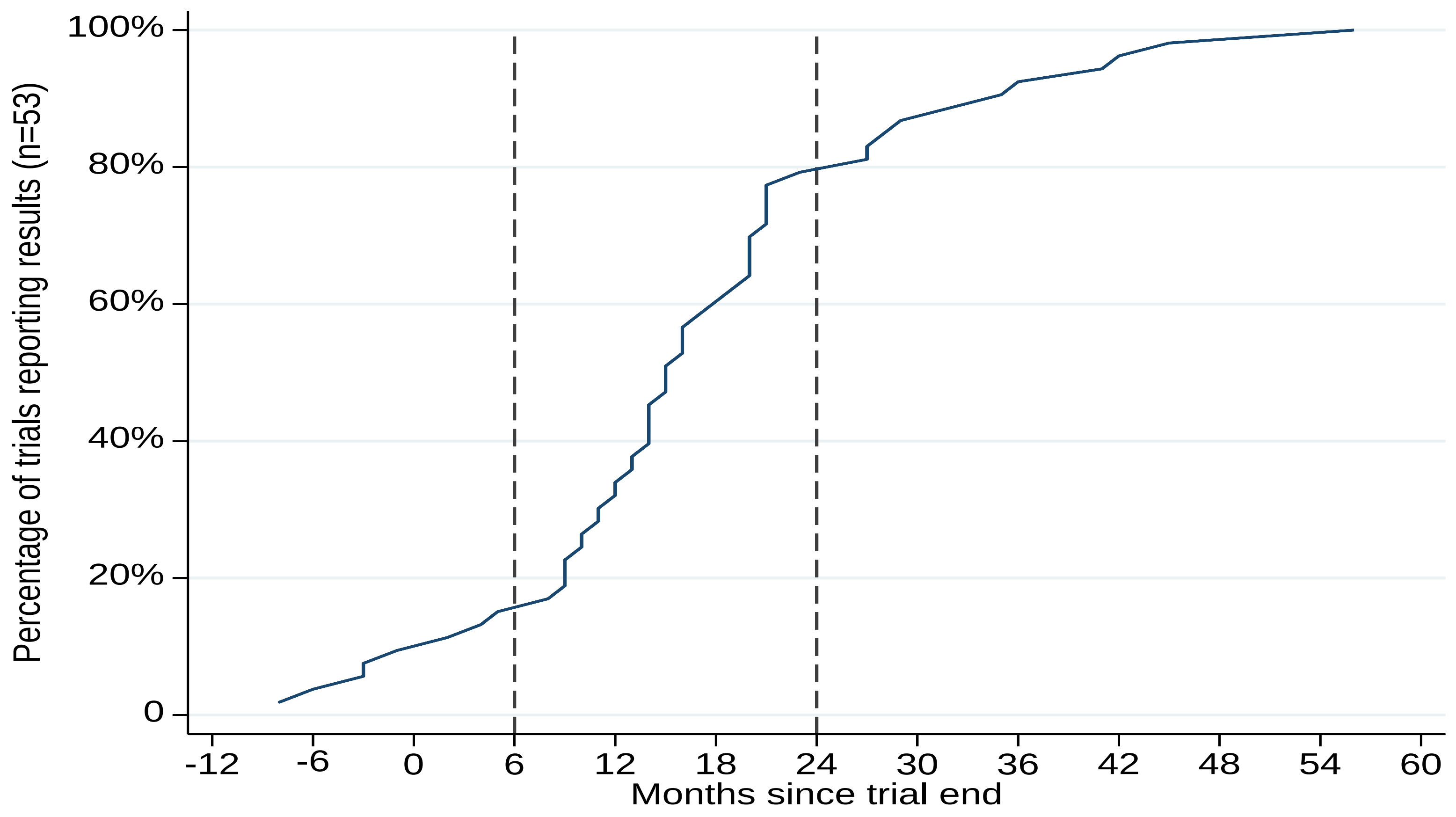

* Nine completed trials that have not yet reported findings were excluded

Figure 4 Reporting (or publication) of main results by year when award ended

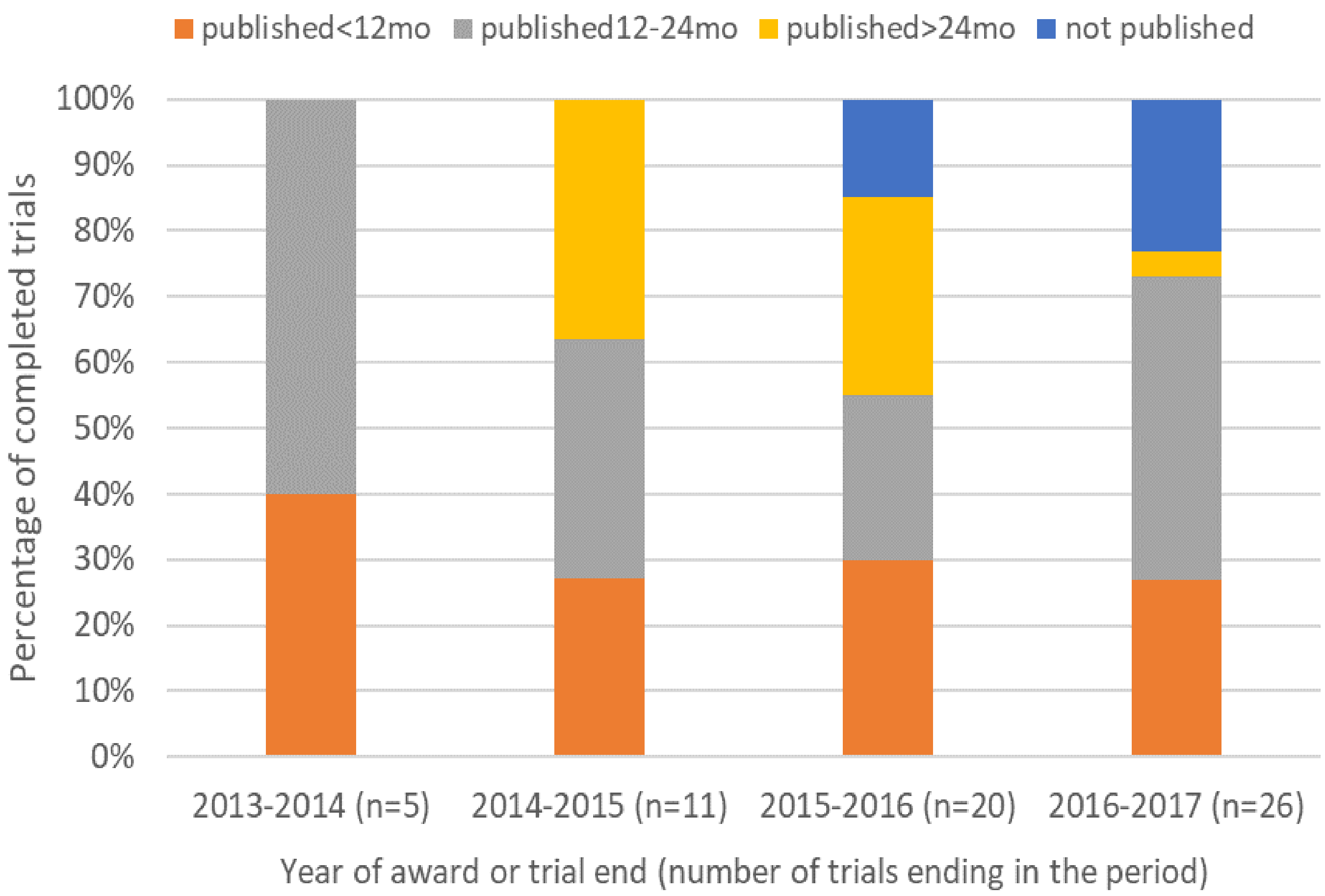

SUPPLEMENTARY FILE 1: POLICY TIMELINE

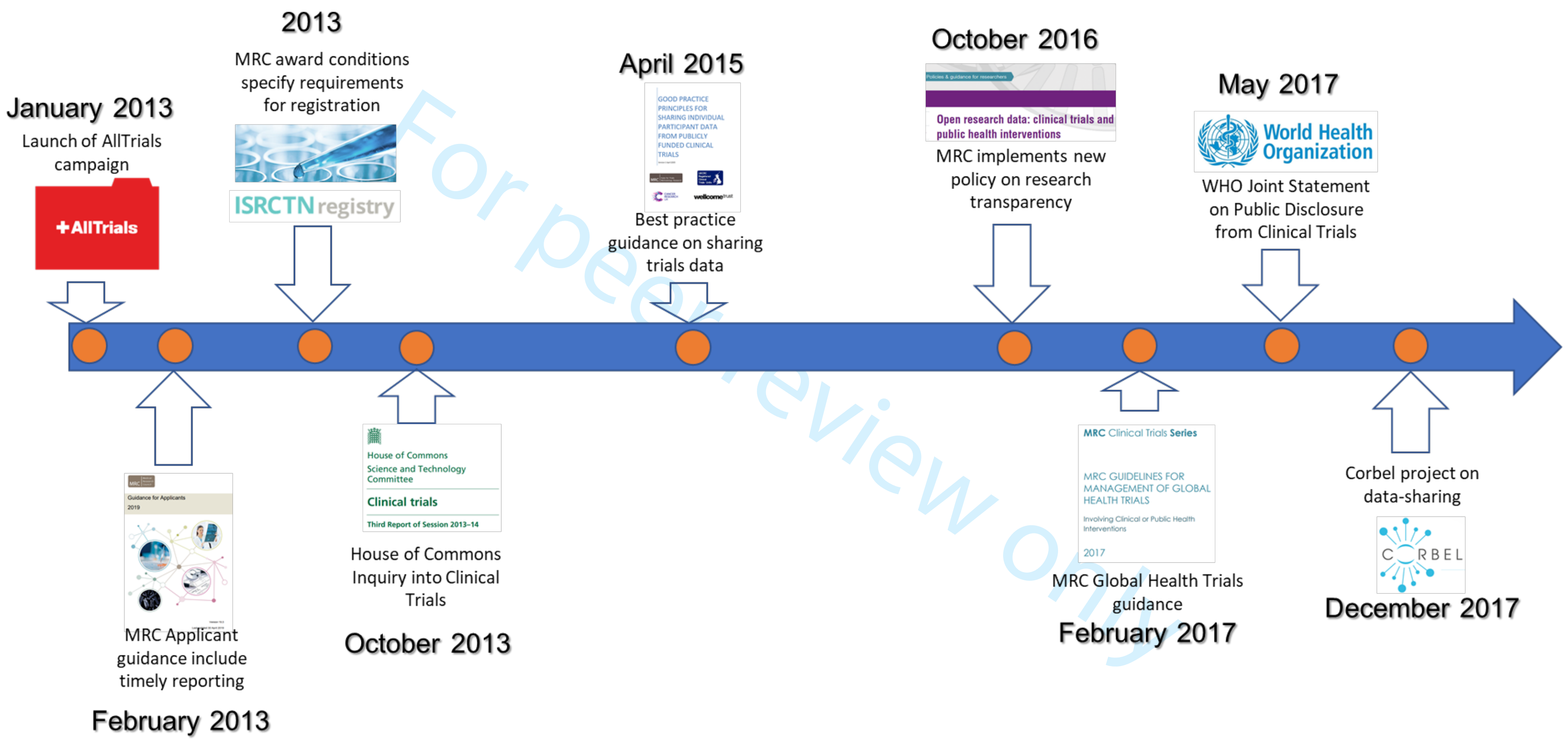

For peer review only

SUPPLEMENTARY FILE 2: SEARCH STRATEGY

Original Search Strategy:

1. Grant application/proposal classification includes any of the following keywords:
    a. ‘MRC Trial’
    b. ‘Clinical Trial’
    c. HRCS Research Activities AND Classification Name begins with [relevant classification code inserted]
2. Keyword search of ‘Clinical Trial’ on:
    a. Organisation or Department Name
    b. Joint Funder name
    c. Funding Scheme or Call
    d. Grant application:
        i. Technical Summary,
        ii. Lay Summary,
        iii. Impact Summary,
        iv. Aims and Objectives,
        v. Title,
        vi. Keywords.
3. Grant application submitted to:
    a. Board Science Late Phase Trials
    b. Board Science Methodology Research Panel

Concepts and Keywords used in the Revised Search Strategy (on all database fields):

1. Concept: Clinical Trials
    - Clinical Trials
    - Controlled Clinical Trials
    - Randomized Controlled Clinical Trials
    - Randomized Controlled Trials
    - Randomized Clinical Trials
2. Concept: Protocols
    - Clinical Protocols
    - Clinical Research Protocols
    - Treatment Protocols
3. Concept: Trials Monitoring
    - Clinical Trials Data Monitoring Committees
    - Data Monitoring Committees
    - Data and Safety Monitoring Boards
    - Safety Monitoring Boards
    - Phase 1 Clinical Trials
4. Concept: Phase I Trials
    - Phase I Clinical Trials
    - Phase 1 Clinical Trials
    - FDA Phase I Evaluation Studies
    - FDA Phase 1 Evaluation Studies
    - Human Microdosing Trials
    - FDA Phase 1 Drug Evaluation
    - FDA Phase I Drug Evaluation
5. Concept: Phase II Trials

For peer review only

- Phase II Clinical Trials
- FDA Phase II Evaluation Studies
- FDA Phase 2 Evaluation Studies
- FDA Phase II Drug Evaluation
- FDA Phase 2 Drug Evaluation

6. Concept: Phase 3 Clinical Trials
    - Phase 3 Clinical Trials
    - Phase III Clinical Trials
    - FDA Phase III Evaluation Studies
    - FDA Phase 3 Evaluation Studies
    - FDA Phase III Drug Evaluation
    - FDA Phase 3 Drug Evaluation
7. Concept: Phase 4 Clinical Trials
    - Phase 4 Clinical Trials
    - Phase IV Clinical Trials
    - FDA Phase IV Drug Evaluation
    - FDA Phase 4 Evaluation Studies
    - FDA Phase IV Evaluation Studies
    - FDA Phase 4 Drug Evaluation
8. Concept: Cohort Studies
    - Cohort studies
    - Closed Cohort Studies
    - Incidence Studies
    - Cohort Analysis
    - Historical Cohort Studies
    - Concurrent Studies
9. Concept: Control Groups
    - Control Groups
10. Concept: Crossover Design
    - Crossover Design
    - Cross-Over Trials
    - Crossover Trials
    - Cross-Over Design
    - Crossover Studies
11. Concept: Double-Blind Method
    - Double-Blind Method
    - Double-Masked Method
    - Double-Blind Study
    - Double-Masked Study
12. Concept: Feasibility Studies
    - Feasibility Studies
13. Concept: Informed Consent
    - Informed Consent
14. Concept: Intervention Studies
    - Intervention Studies
15. Concept: Mass Screening
    - Mass Screening
16. Concept: Multicenter Studies
    - Multicenter Studies
    - Multicenter Trials

For peer review only

17. Concept: Patient Compliance
    - Patient Compliance
    - Patient Non-Adherence
    - Patient Non-Compliance
    - Patient Nonadherence
    - Patient Cooperation
    - Patient Adherence
    - Patient Noncompliance
18. Concept: Placebos
    - Placebos
    - Sham Treatment
19. Concept: Research Design
    - Data Reporting
    - Research Technics
    - Data Adjustment
    - Research Proposal
    - Data Quality
    - Matched Groups
    - Research Techniques
    - Experimental Designs
    - Problem Formulation
    - Research Methodology
    - Scoring Methods
    - Error Sources
20. Concept: Single-Blind Method
    - Single-Blind Study
    - Single-Masked Method
    - Single-Masked Study
21. Concept: Investigational Therapies
    - Innovative Therapies
    - Experimental Therapies
    - Investigational Therapies
    - Investigational Treatments

For peer review only
